# Genomic Organization and Expression Demonstrate Spatial and Temporal *Hox* Gene Colinearity in the Lophotrochozoan *Capitella* sp. I

**DOI:** 10.1371/journal.pone.0004004

**Published:** 2008-12-23

**Authors:** Andreas C. Fröbius, David Q. Matus, Elaine C. Seaver

**Affiliations:** Kewalo Marine Lab, Pacific Biosciences Research Center, University of Hawaii, Honolulu, Hawaii, United States of America; University College Dublin, Ireland

## Abstract

*Hox* genes define regional identities along the anterior–posterior axis in many animals. In a number of species, *Hox* genes are clustered in the genome, and the relative order of genes corresponds with position of expression in the body. Previous *Hox* gene studies in lophotrochozoans have reported expression for only a subset of the *Hox* gene complement and/or lack detailed genomic organization information, limiting interpretations of spatial and temporal colinearity in this diverse animal clade. We studied expression and genomic organization of the single *Hox* gene complement in the segmented polychaete annelid *Capitella* sp. I. Total genome searches identified 11 *Hox* genes in *Capitella*, representing 11 distinct paralog groups thought to represent the ancestral lophotrochozoan complement. At least 8 of the 11 *Capitella Hox* genes are genomically linked in a single cluster, have the same transcriptional orientation, and lack interspersed non-*Hox* genes. Studying their expression by situ hybridization, we find that the 11 *Capitella Hox* genes generally exhibit spatial and temporal colinearity. With the exception of *CapI-Post1*, *Capitella Hox* genes are all expressed in broad ectodermal domains during larval development, consistent with providing positional information along the anterior–posterior axis. The anterior genes *CapI-lab*, *CapI-pb*, and *CapI-Hox3* initiate expression prior to the appearance of segments, while more posterior genes appear at or soon after segments appear. Many of the *Capitella Hox* genes have either an anterior or posterior expression boundary coinciding with the thoracic–abdomen transition, a major body tagma boundary. Following metamorphosis, several expression patterns change, including appearance of distinct posterior boundaries and restriction to the central nervous system. *Capitella Hox* genes have maintained a clustered organization, are expressed in the canonical anterior–posterior order found in other metazoans, and exhibit spatial and temporal colinearity, reflecting *Hox* gene characteristics that likely existed in the protostome–deuterostome ancestor.

## Introduction

*Hox* genes have represented one of the major paradigms of developmental biology for nearly three decades (reviewed in [1], [2]). These homeodomain genes encode transcription factors that, via regulation of various downstream genes, are capable of imprinting positional identities on to distinct body domains along the anterior-posterior axis of the animal. Among the most fascinating characteristics of these genes are that *Hox* genes are organized into clusters in the genome in some animals, and there is a precise relationship between the order of genes in the cluster and the relative postions of expression domains along the anterior–posterior axis of the body, a phenomenon called spatial colinearity (reviewed in [3], [4]). *Hox* genes positioned at the 3′ end of the cluster are expressed and pattern the anterior end of the embryo while *Hox* genes at the 5′ end of the cluster pattern the posterior of the embryo. In addition, in several animals (reviewed in [4]), *Hox* genes exhibit temporal colinearity, and the temporal order of initiation of expression reflects the order of *Hox* genes in the cluster (e. g., “anterior” genes are expressed earlier than genes with posterior expression domains).

*Hox* genes have been isolated from all major clades of bilaterians and cnidarians that have been studied. The species-specific repertoire, genomic organization, presence or absence of clusters and, in the case of vertebrates, numbers of clusters, and their deployment have formed the basis of models of animal body plan evolution and diversification [5], [6]. However, these models have been largely based on studies limited to deuterostomes and ecdysozoans, limiting the inferences that can be made about *Hox* genes in the protostome/deuterostome ancestor. In a recent review [3], Duboule points out that the discovery of *Hox* gene clusters in flies and mice quickly led to the assumption that all other animals have such clusters, yet direct demonstration of genomic linkage among *Hox* genes is far more limited than is generally appreciated. In addition, in animals whose genomes contain a *Hox* gene cluster, there is significant variation in the level of organization of the cluster. In vertebrates, *Hox* genes within a cluster share the same transcriptional orientation, and non-*Hox* genes are not interspersed among them [3]. In contrast, in the ANT-C complex of *Drosophila* [7] and the *Hox* cluster of sea urchins [8], *Hox* genes are present in both transcriptional orientations, and the *Drosophila* ANT-C complex also has non-*Hox* genes present within the cluster. Other deuterostomes such as the larvacean *Oikopleura dioica* do not have clustered *Hox* genes [9]. Thus, there is significant variation in genomic organization of *Hox* genes even within deuterostomes.

Far less is known about *Hox* genes in lophotrochozoans. Although gene fragments have been recovered from a wide range of lophotrochozoans such as nemerteans, molluscs, flatworms, and annelids (including echiurans), expression data for Hox genes has been reported for many fewer species. Such expression studies include the polychaete annelids *Chaetopterus variegates* [10], *Platynereis dumerilii*, and *Nereis virens* [11]; the leeches *Helobdella robusta*, *Helobdella triseralis* [12], and *Hirudo medicinalis* [13]–[16]; the squid *Euprymna scolopes* [17]; the gastropod *Haliotis asinina* [18]; and the planarian *Dugesia* [19]–[21]. Most of these studies are incomplete, and expression for the full *Hox* gene complement within a single species has not been determined. Genomic organization data is even more limited. In the platyhelminth parasite *Schistosoma mansoni*, fluorescent in situ hybridization analysis of four *Hox* genes shows localization to two different chromosomes, supporting the conclusion that *S. mansoni* does not have a single *Hox* cluster [22]. In the nemertean *Lineus sanguineus*, preliminary analysis shows hybridization of two *Hox* genes to the same fragment of genomic DNA by southerm blot analysis [23]. Thus, for an expected complement of at least 10 *Hox* genes in lophotrochozoans [24], evidence for more than two linked *Hox* genes has not yet been reported. The lack of genomic organization and expression data within a single lophotrochozoan species has seriously limited analysis of gene order and determination of spatial or temporal colinearity.

*Capitella* sp. I is a segmented polychaete annelid with a variable number of adult body segments. This semi-direct developer generates 13 segments during larval life. Following a short nonfeeding pelagic phase, animals undergo metamorphosis, which is accompanied by body elongation and loss of the ciliated prototroch, telotroch, and neurotroch. *Capitella* sp. I juveniles immediately commence feeding and continue to add segments by posterior addition. The adult body plan has distinct thoracic and abdominal regions, although within each region, segments appear morphologically similar. The gradual formation of more than a dozen segments during larval development in a several-day period, the simple induction of metamorphosis, and the addition of segments from a posterior growth zone during juvenile life enabled us to study temporal and spatial *Hox* gene expression at multiple life history stages and during two different modes of segmentation not possible in some other polychaete models.

In this study, we present detailed genomic linkage data of the first lophotrochozoan *Hox* cluster and expression patterns for these *Hox* genes from the polychaete annelid *Capitella* sp. I during larval and juvenile stages. We address the following questions: (1) Do the *Capitella* sp. I *Hox* genes exhibit spatial and temporal colinearity? (2) Are expression patterns observed consistent with possible roles in establishing positional identity? (3) Is there a correlation between *Hox* gene expression with morphological features or boundaries (e.g., thoracic–abdominal boundary)? (4) Are *Hox* genes involved in patterning of the segments added by a localized posterior growth zone during adult life? (5) Are there commonalities in *Hox* gene expression among annelids? (6) Are features of *Hox* gene expression in annelids conserved with other metazoans?

## Methods

### *Capitella* sp. I Culture

A colony of *Capitella* sp. I was maintained using culture methods developed by [25] as described [26]. Embryos and larvae [26] and juveniles [27] of *Capitella* were collected as previously described.

### Cloning of *Capitella* sp. I *Hox* Genes

Fragments of *Hox* gene orthologs were isolated from either genomic DNA (gDNA) or an excised lambda ZAPII library generated from mixed embryonic and larval stages of *Capitella* sp. I, and several degenerate primer sets corresponding to the conserved homeodomain were used in degenerate PCR amplifications. 117-bp fragments of the central class *Hox* genes *CapI-Dfd*, *CapI-Scr*, *CapI-lox5*, *CapI-lox4*, and *CapI-lox2* were amplified with the primers ELEKEF (5′-GARYTNGARAARGARTT-3′) and WFQNRR (5′-CKNCKRTTYTGRAACCA-3′). A 141-bp fragment of *CapI-lb* was recovered using the primers NFTNKQLT (5′-AAYTTYACHAAYAARCARYTSAC-3′) and WFQNRR, and *CapI-Hox3* (155 bp) with KLARTAYT (5′-AAGCTTGCCMGNACNGCNTAYAC-3′) and WFQNRR. *CapI-pb* (108 bp) was recovered by semi-nested PCR with the primers ARTAYT (5′-GCNMGNACNGCNTAYAC-3′) and WFQNRR, followed by ARTAYT and IAASLD (5′-TCSARNGARGCRGCDATC-3′) with 1∶100 diluted product of the first round as template for a second PCR reaction. 159-bp fragments of *CapI-Post1* and *CapI-Post2* were isolated using the primers RKKRKPY (5′-MGIAARAARMGIAARCCNTA-3′) and WFQNRR.

Additional sequence for each *Hox* gene was obtained using gene specific primers (sequences available upon request) in RACE (rapid amplification of cDNA ends) reactions in combination with either mixed embryonic/larval cDNA library as a template or RACE using the SmartRACE Kit (BD Biosciences). Fragments were conceptually spliced together and submitted to GenBank as composite transcripts with the following accession numbers: *CapI-lab*, EU196537; *CapI-pb*, EU196538; *CapI-Hox3*, EU196539; *CapI-Dfd*, EU196540; *CapI-Scr*, EU196541; *CapI-lox5*, EU196542; *CapI-Antp*, EU196547; *CapI-lox4*, EU196543; *CapI-lox2*, EU196544; *CapI-Post2*, EU196545; and *CapI-Post1*, EU196546. Predicted open reading frames (ORFs) were identified using MacVector. All degenerate and RACE fragments were cloned into the pGEM-T_easy_ vector (Promega) and sequenced at the University of Hawaii sequencing facility or Macrogen Inc. (South Korea).

### Linkage Analysis

The *Capitella* sp. I genome (v1.0; Joint Genome Institute, Department of Energy) was searched using nucleotide sequences of *Hox* genes isolated by degenerate PCR. Spidey (http://www.ncbi.nlm.nih.gov/spidey) was used to compare genomic and previously isolated cDNA sequences to determine transcription units of *Capitella* sp. I *Hox* genes. Lengths given for introns and exons are based on these comparisons. Additional exons or introns might not have been identified due to incomplete RACE products. Scaffold data were analyzed by Genscan, a gene prediction algorithm (http://genome.dkfz-heidelberg.de/cgi-bin/GENSCAN/genscan.cgi), and predicted ORFs were compared with sequences in GenBank by blastp and tblastn (http://www.ncbi.nlm.nih.gov/BLAST).

### Orthology Assignments and Phylogenetic Analyses

Putative orthology assignments of *Capitella* sp. I *Hox* sequences were made via BLASTX searches of the GenBank database from the National Center for Biotechnology Informaiton (NCBI). An amino acid alignment of the 60–amino acid (AA) homeodomain and 12 AAs directly flanking the 3′ end of the homeodomain was generated, and includes representative sequences from acoels and nemertodermatid flatworms (*Symsagittifera roscoffensis*, *Nemertoderma westbladii*), chaetognaths, an ecdysozoan (*Tribolium castaneum* [beetle]), lophotrochozoans (*Nereis virens* and *Capitella* sp. I [annelid] and *Euprymna scolopes* [mollusk]), and a deuterostome (*Branchiostoma floridae* [cephalochordate]). Additional lophotrochozoan paralog group 7 (PG7) sequences included are from the brachiopod *Lingula anatine* and the nemertean *Lineus sanguineus*. Bayesian phylogenetic analyses were conducted with MrBayes 3.1.2 [28] using a mixed amino acid model with gamma, which selected RtRev with a 100% posterior probability with 3,000,000 generations sampled every 100 generations with 4 chains over 4 independent runs. A summary tree was produced from the final 23,000 trees representing 2,300,000 stationary generations per run, and 92,000 trees representing 9,200,000 stationary generations for the consensus tree. In addition, neighbor joining (NJ) (using mean AA distances) was conducted with PAUP* v4.0b10 [29]. ProtTest [30] selected the rtrev+G model, which was used for maximum likelihood (ML) analyses conducted using RAXML v2.2.1 [31]. An initial search was conducted in RAXML v2.2.1 using 500 searches with the rtrev+G model. A consensus of this search is shown in Figure S3. ML bootstrap analysis was also conducted using RAXML v2.2.1 with 1,000 iterations. *Hox* genes were assigned to paralog groups using the same methodology as Kourakis et al. [32] and Matus et al. [33], and as discussed in Balavoine et al. [24]. Based on multiple methods of phylogenetic analyses, *Capitella Hox* genes cluster with representative orthologous genes from other taxa. For example, *CapI-lab* clusters with chaetognath, arthropod, annelid, cephalochordate, mollusc, and acoel *Hox1*/*lab* genes with 100% posterior probability, all belonging to PG1. Alignment is presented in Figure S1 and available upon request.

### Whole Mount In Situ Hybridization

Stages 2–4 embryos were permeabilized by treatment with 0.5 M sucrose/0.125 M sodium citrate for 3 min, and larvae and juveniles were relaxed in 1∶1 0.37 Mol/l MgCl_2_/filtered sea water (FSW) for 15 min prior to fixation in 3.7% formaldehyde in FSW at 4°C overnight. All stages were washed in phosphate-buffered saline (PBS) 3 times, dehydrated, and stored in methanol at −20°C. The whole mount in situ hybridization protocol has been published previously [34]. Linear templates for probe synthesis were generated by PCR with oligonucleotides against SP6 and T7 promotor regions. Digoxigenin-labeled riboprobes were generated using the MEGAscript High Yield Transcription Kit (Ambion, Austin, Texas, United States of America) in the presence of 11-dig-UTP (Roche). Riboprobes were hybridized to tissue in hybridization buffer (50% formamide, 5× SSC [pH 4.5], 50 µg/ml heparin, 0.1% Tween-20, 1% SDS, and 100 µg/ml sheared salmon sperm DNA) at 65°C for 72 h, followed by increasingly stringent washes of SSC to 0.05× SSC. All riboprobes were used at working concentrations of 3 ng/µl. Riboprobes were generated from the following fragments: 810-bp 5′ RACE fragment for *CapI-lb*, 1,023-bp 5′ RACE fragment for *CapI-pb*, 1,337-bp 5′ RACE fragment for *CapI-Hox3*, 709-bp 5′ RACE fragment for *CapI-Dfd*, 1,116-bp 5′ RACE fragment for *CapI-Scr*, 947-bp 5′ RACE fragment for *CapI-lox5*, 662-bp 5′ RACE fragment for *CapI-Antp*, 889-bp 5′ RACE fragment for *CapI-lox2*, 1,090-bp 5′ RACE-fragment for *CapI-Post2*, and a 585-bp 5′ RACE fragment, a 532-bp 3′ RACE fragment, and an 883-bp combined fragment were tested for *CapI-Post1* (wells containing *CapI-Post1* probes were extensively overstained). Specimens were analyzed using differential interference contrast (DIC) optics on a Zeiss Axioskop Plus microscope, and digital photomicrographs were captured with a Nikon Coolpix 4500 digital camera (4.0 megapixel). The detailed protocol is available upon request.

## Results

### Isolation of *Hox* Gene Orthologs from *Capitella* sp. I

To isolate *Hox* genes from *Capitella* sp. I, a mixed-stage embryonic and larval library was screened by PCR using degenerate primers designed to conserved regions of previously isolated lophotrochozoan *Hox* genes for specific *Hox* classes. Fragments of 10 *Hox* genes were recovered. Additional sequence was retrieved by RACE using gene-specific primers and by BLAST searches of genomic trace files. An eleventh *Capitella* sp. I *Hox* gene, *CapI-Antp*, was identified by a directed search of the *Capitella* sp. I genome (Joint Genome Institute [JGI]). Lengths of recovered fragments, predicted ORFs, GenBank accession numbers, paralogy groups, and protein ID numbers from the annotated *Capitella* sp. I genome project (JGI) are shown in Table 1.

**Table 1:** *Capitella* sp. I *Hox* genes.

Paralogy group	Gene name	5′ RACE fragment	3′ RACE fragment	Composite transcript	Predicted ORF (a.a.)	Genbank accession #	Protein ID (JGI)
PG1	CapI-lab	810 bp	781 bp	1562 bp	297	EU196537	219807
PG2	CapI-pb	971 bp	-	1006 bp	238	EU196538	94911
PG3	CapI-Hox3	1337 bp	2154 bp	3081 bp	407	EU196539	219808
PG4	CapI-Dfd	709 bp	737 bp	1046 bp	181	EU196540	149640
PG5	CapI-Scr	1116 bp	779 bp	1676 bp	198	EU196541	168122
PG6	CapI-lox5	947 bp	1111 bp	2037 bp	285	EU196542	168125
PG7	CapI-Antp	662 bp	-	1481 bp	105	EU196547	94879
PG8	CapI-lox4	818 bp	817 bp	1331 bp	258	EU196543	94902
PG8	CapI-lox2	908 bp	1287 bp	1768 bp	255	EU196544	225442
PG9-14	CapI-Post2	1038 bp	915 bp	1538 bp	285	EU196545	126009
PG9-14	CapI-Post1	585 bp	532 bp	736 bp	177	EU196546	70154

Protein ID numbers are from the Joint Genome Institute annotated genome of *Capitella* sp. I (http://genome.jgi-psf.org/Capca1/Capca1.home.html). ‘-’ indicates that a RACE fragment was not recovered.

### Orthology Assignments of *Capitella* sp. I *Hox* Genes

**Figure 1:**
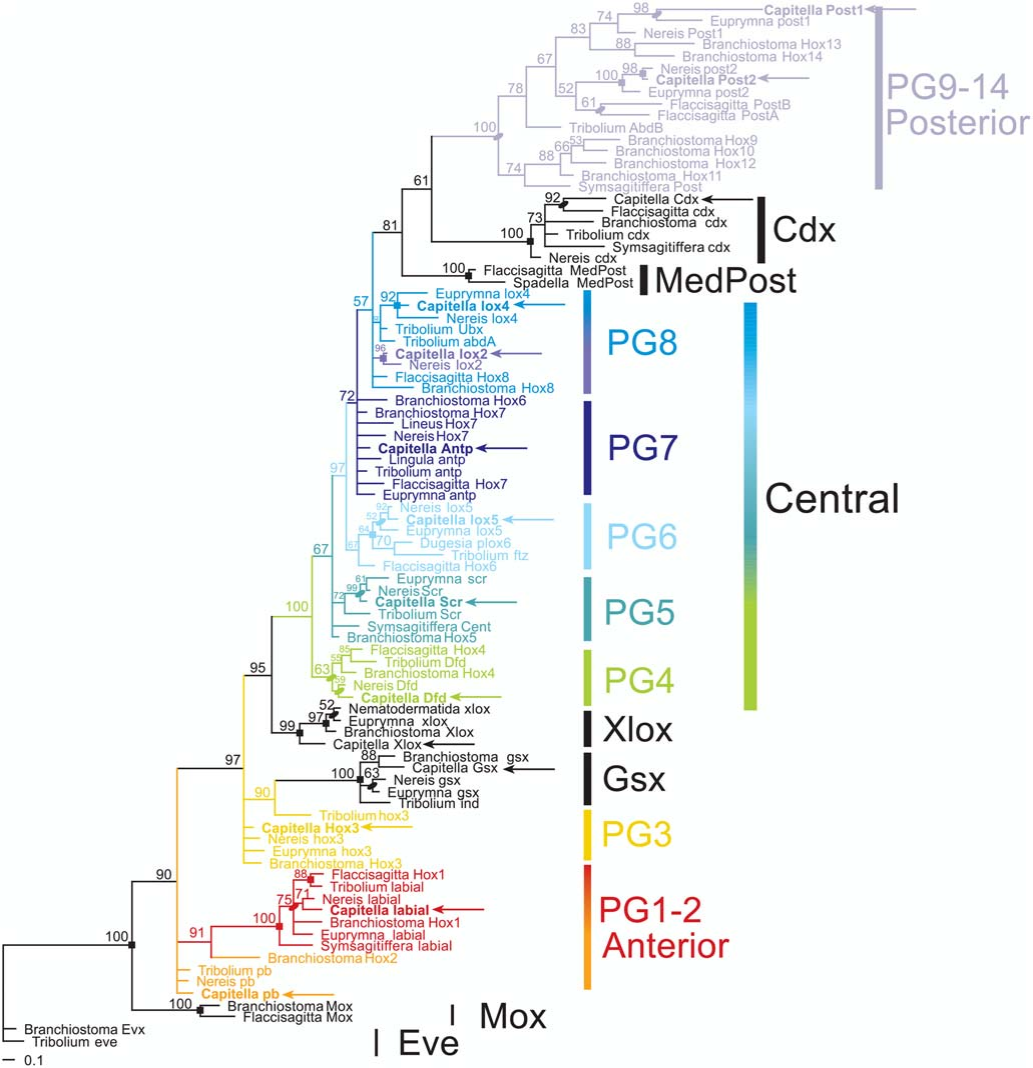
Orthology assignments for *Capitella* sp. I *Hox* genes. Bayesian phylogenetic analysis was conducted using an alignment of the 60–amino acid homeodomain and the 12 amino acids immediately 3′ of the homeodomain for representative bilaterian *Hox* and *Parahox* genes. *Capitella* sp. I possesses orthologs for each *Hox* paralogy group (PG). Sequences from representative species include *Tribolium castaneum* (ecdysozoan); *Nereis virens*, *Euprymna scolopes*, and *Capitella sp.* I (lophotrochozoan); *Flaccisagitta enflata* and *Spadella cephaloptera* (chaetognath); *Symsagitiffera roscoffensis* and *Nemertoderma westbladii* (acoelomorphs); *Branchiostoma floridae* (deuterostome); and additional PG7 lophotrochozoan sequences (see Methods). *Capitella sp.* I sequences are delimited by arrows; *Capitella* sp. I *Hox* sequences are shown in bold. Numbers above branches indicate Bayesian posterior probabilities. Ovals delimit bootstrap support >50 at a node from either neighbor-joining (NJ) or maximum likelihood (ML) analyses, while squares show bootstrap support >50 from both NJ and ML analyses. Colors are unique to each *Hox* PG. Individual NJ and ML bootstrap consensus trees are shown in Figures S2 and S3, respectively.

The *Capitella* sp. I genome possesses definitive members of all four classes of *Hox* genes proposed to be present in the bilaterian ancestor [32], [35], including anterior, PG3, central, and posterior class genes (Figure 1). *Hox* genes can be classified both by phylogenetic analyses and also the presence of diagnostic AA motifs found within and flanking the highly conserved 60-AA homeodomain [24], [36], [37]. Phylogenetic analyses suggest a similar *Hox* gene complement in *Capitella* as is found in other lophotrochozoans (molluscs [17], [18], brachiopods [37], nemerteans [23], platyhelminths [38], and other annelids [11], [32], [39]). At least 10 distinct *Hox* genes were previously proposed to comprise the lophotrochozoan complement [24], and we isolated 11 *Capitella* sp. I *Hox* genes. A total of 11 *Hox* genes have also been reported for *Nereis virens* [11]. Phylogenetic analyses suggest that these 11 genes can each be assigned to distinct paralog groups (Figure 1), including two anterior class genes (*Labial/Hox1* and *Proboscipedia/Hox2*), a single PG3 gene (*Hox3/zen*), six central class genes (PG4: *Deformed/Hox4*; PG5: *Sex combs reduced/Hox5*; PG6: *Lox5/fushitarazu*; PG7: *Antennapedia*; and PG8: *Lox4/Lox2/Ultrabithorax/Abdominal-A*) and two posterior genes (PG9-14: *Post1/Post2/AbdB*). *Capitella* sp. I has a definitive *Antp*/PG7 gene, which clusters with other lophotrochozoan, ecdysozoan, and deuterostome central class genes, including the ecdysozoan *Tribolium antp* gene (Figure 1). Notably, *CapI-Antp* does not cluster with lophotrochozoan *lox5*, *lox4*, or *lox2* genes. This suggests that *Capitella Antp* is a member of the PG7 genes, which includes other previously identified orthologs from another polychaete (*Nereis Hox7*), a brachiopod (*Lingula Antp*), and a nemertean (*Lineus Hox7*); ecdysozoan *Antp* genes; a chaeotgnath *Hox* gene (*Flaccisagitta Hox7*); and possibly deuterostome *Hox6* and *Hox7* genes.

### The *Capitella* sp. I *Hox* Cluster

**Figure 2:**
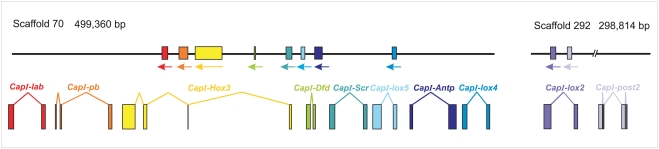
Genomic organization of the *Capitella* sp. I Hox cluster. A total of 11 *Capitella* sp. I *Hox* genes are distributed among three scaffolds. Black lines depict two scaffolds, which contain 10 of the *Capitella* sp. I *Hox* genes. The eleventh gene, *CapI-Post1*, is located on a separate scaffold surrounded by ORFs of non-*Hox* genes (unpublished data). No predicted ORFs were identified between adjacent linked *Hox* genes. Transcription units are shown as boxes denoting exons, connected by lines that denote introns. Transcription orientation is denoted by arrows beneath each box. Color coding is the same as that used in Figure 1 for each ortholog.

*Capitella* sp. I *Hox* genes are located on three contigs: *CapI-lab*, *CapI-pb*, *CapI-Hox3*, *CapI-Dfd*, *CapI-Scr*, *CapI-lox5*, *CapI-Antp*, and *CapI-lox4* are all on scaffold 70 and span 243 kbp (Figure 2). *CapI-lox2* and *CapI-Post2* are located on scaffold 292 spanning 21.6 kbp. Predicted non-*Hox* genes flank one side of *CapI-lab* on scaffold 70 and *CapI-Post2* on scaffold 292. In contrast, no genes with similarity to previously characterized genes were identified between adjacent *Hox* genes. The lack of predicted ORFs within the 100-kb 5′ of *CapI-lox4* (from *CapI-lox4* to the end of the scaffold) and 23.4 kb 3′ of *CapI-lox2* (from *CapI-lox2* to the end of the scaffold) is consistent with a larger *Hox* cluster in the genome, although we do not currently have direct evidence of linkage between these two scaffolds. If scaffolds 70 and 292 are linked, the cluster would span at least 345 kb. *CapI-Post1* is on scaffold 33, and predicted non-*Hox* genes were identified in proximity to both 5′ and 3′ of this transcription unit, suggesting that the *Post1*-ortholog is not part of the *Capitella Hox* cluster.

*CapI-lab* is located at the 3′ end of the *Hox* cluster (Figure 2). Its 6-kb transcription unit is composed of two exons (617 and 872 bp in size) separated by an 4,569 bp intron. The *CapI-pb* gene, located 11.6 kb 5′ of *CapI-lab*, contains three exons (544, 254, and 173 bp). The first two are separated by a large intron of 7.7 kb, and the small second intron (513 bp) is located within the homeobox. *CapI-Hox3* is located 7.6 kb 5′ of *CapI-pb*. This 27.8-kb transcription unit contains three introns (16.6, 6.7, and 1.4 kb) separating four exons of 385, 46, 563, and 2,070 bp. *CapI-Dfd* has a small transcription unit of 1.5 kb, with two exons of 395 and 651 bp, separated by a 439-bp intron, and is located 33.3 kb 5′ of *CapI-Hox3*. The 6.2-kb transcription unit of *CapI-Scr* is 31.4 kb upstream of *CapI-Dfd* and consists of two exons of 612 and 876 bp, separated by a 4.7-kb intron. The *CapI-lox5* transcript is composed of two exons (606 and 1,397 bp) and a single intron of 2 kb, located 9.4 kb 5′ of *CapI-Scr*. *CapI-Antp* is located about 17.2 kb 5′ of *CapI-lox5*. The transcription unit of *CapI-Antp* contains two exons of 640 and 841 bp separated by a 6-kb intron. The transcription unit of *CapI-lox4* is 4.5 kb and contains a single intron of 3.2 kb separating two exons of 520 and 756 bp. *CapI-lox4* is located 90.4 kb 5′ of *CapI-lox5*. On scaffold 70, the 5.5-kb *CapI-lox2* transcript contains a single intron of 3.8 kb separating two exons of 1,169 and 599 bp. *CapI-Post2* is located 11.4 kb 5′ of *CapI-lox2* and has four exons (602, 143, 582, and 158 bp) and three introns (122, 2,905, and 147 bp).

### *Capitella* sp. I Development

Development of *Capitella* sp. I has been described previously [40]–[43], and occurs within a parental brood tube. Following gastrulation and elongation of the embryo along the anterior–posterior axis, two anterior epidermal thickenings initiate formation of the bilobed brain, and an anterior invagination at the ventral midline marks the position of the mouth at late stage 3. By the beginning of stage 4, two trochal bands, the prototroch and telotroch, are formed. At stage 5, the first morphologically defined segments are evident, and within the next 24 h, 10 segments appear with an anterior–posterior temporal progression [26]. These 10 segments arise from the ventro–lateral region of the larva and expand dorsally around the body circumference over time [44]. The presumptive segmental tissue is called the “bauchplatten” or “belly plates” by Eisig [40]. The medial side of the belly plates on the ventral side of the larva contributes to the ventral nerve cord (VNC). Additional segments are added from a posterior growth zone, resulting in a total of 13 larval segments (nine thoracic segments [T1–T9] and four abdominal segments [A1–A4]). *Capitella* sp. I has a nonfeeding larva, and adult gut morphogenesis occurs during late larval stages, resulting in a distinct pharynx, esophagus, midgut, and hindgut [45]. By stage 9, *Capitella* larvae are competent to undergo metamorphosis, triggered by stimuli in the sediment. During metamorphosis, the body loses its trochal bands, elongates, initiates feeding, and becomes limited to a benthic lifestyle. Like other polychaetes, *Capitella* is capable of generating new segments at multiple life history stages, and juveniles continue to grow by posterior addition of segments. The body has a distinct thoracic region of nine segments and an abdominal region of comparatively thinner segments with approximately 55 segments in mature adults.

### Larval Expression of Anterior Class *Hox* genes *CapI-lab* and *CapI-pb*

Temporal and spatial expression of all *Hox* genes was analyzed by whole mount in situ hybridization from early larval stages (stage 4) to 3 d following metamorphosis. Specificity of the probes was confirmed by processing control larvae and juveniles without probe or with sense probes. No staining was observed in these controls (not shown).

**Figure 3:**
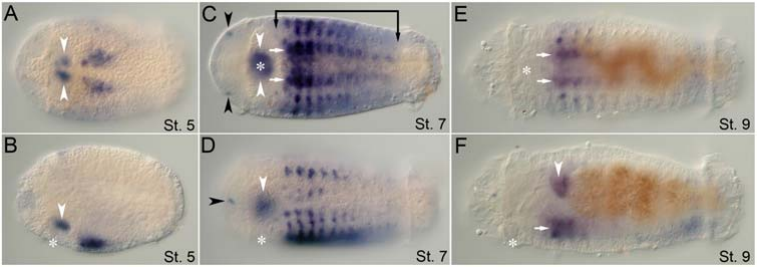
Expression of *CapI-lab* during larval development as analyzed by in situ hybridization. Anterior is to the left for all panels. Stages are indicated at bottom right of each panel. (A), (C), and (E) show ventral views; (B), (D), and (F), lateral views. Asterisk marks position of the mouth. (A, B) At stage 5, *CapI-lab* is expressed in the dorsal wall of the stomodeum (white arrowheads) and in bilateral patches of anterior ectoderm. (C, D) *CapI-lab* expression at mid-stage 7 includes two clusters of 2 to 3 cells in head ectoderm (black arrowheads), dorsolateral and ventrolateral ectodermal domains and foregut tissue (white arrowheads). Bracket denotes anterior and posterior borders of *CapI-lab* expression in the mid-body. White arrows mark staining in the VNC. (E, F) Expression in the VNC (white arrows) and the posterior of the esophagus (white arrowhead) persists to the end of larval development (stage 9).

During larval development, the *CapI-lab* transcript is expressed in a number of distinct tissues. Weak expression is initially detectable at stage 4 in bilateral anterio–medial domains within the presumptive segmental tissue. This expression appears prior to segmentation (unpublished data). At stage 5, *CapI-lab* is expressed in two discrete areas: a pair of closely positioned patches in the dorsal wall of the stomodeum, and continued expression in the segmental tissue in the region of the VNC (thoracic segments, T2–T4; Figure 3A and 3B). During mid-larval stages after the brain is well developed (stage 7), *CapI-lab* is also expressed in two small bilateral clusters of 2 to 3 cells each in the head epidermis, possibly head sensory neurons (Figure 3C and 3D). Foregut expression persists and increases in area. *CapI-lab* expression expands to include all segments, with highest levels in T2 and T3, but is absent from the posterior growth zone. At this stage, segmental *CapI-lab* expression in the epidermis is in discrete ventrolateral and dorsolateral patches, with additional lateral patches in the four anterior-most thoracic segments (Figure 3C and 3D). At stage 8, *CapI-lab* expression is downregulated in all tissues. Once the larva is competent to undergo metamorphosis at stage 9, *CapI-lab* expression is no longer detectable in the head (Figure 3E and 3F). Staining in the VNC is now limited to segments T2 to T5 (Figure 3E). Low levels of epidermal expression are present in the most posterior abdominal segments, increasing in intensity from anterior to posterior. Gut expression is now clearly localized to the esophagus (Figure 3F).

**Figure 4:**
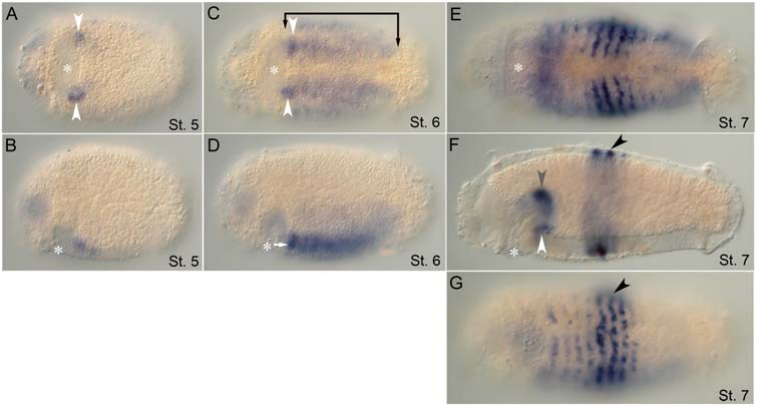
Expression patterns of *CapI-pb* during larval stages. Anterior is to the left for all panels. Stages are indicated at the bottom right of each panel. (A), (C), and (E) show ventral views; (B), (D), (F), and (G), lateral views with ventral down. Asterisk marks the position of the mouth. (A, B) *CapI-pb* expression in two ectodermal clusters, lateral and slightly posterior to the mouth at stage 5 (white arrowheads). (C, D) At the transition from stage 6 to stage 7, *CapI-pb* is expressed in the VNC (white arrow), including prominent expression in the subesophageal ganglion (white arrowheads), and ventro-lateral epidermis of all segments (bracket). (E, F) By late stage 7/early stage 8, the trunk has a segmentally iterated ectodermal stripe pattern (black arrowhead). Additional *CapI-pb* expression appears in the foregut (gray arrowhead), Expression persists in the subesophageal ganglion (white arrowhead). (G) Surface view showing segmentally iterated ectodermal stripes of expression, which is most prominent in T5 to T7 (black arrowhead).

*CapI-pb* expression is initiated at approximately the same time as *CapI-lab* in two small domains lateral and posterior to the mouth (unpublished data). At stage 5, it becomes apparent that this expression is in the subesophageal ganglion (Figure 4A and 4B). At mid-larval stages (late stage 6/early stage 7), additional expression appears in the VNC and in the ventro–lateral epidermis (Figure 4C and 4D). After early stage 7, the expression pattern rapidly changes, and *CapI-pb* becomes expressed in segmentally iterated epidermal stripes by late stage 7 (Figure 4E–4G). Each “stripe” is approximately 3 to 4 cells wide, centered within each segment, and discontinuous in places along its length with no connection at the ventral midline (Figure 4E and 4G). Although detectable in all segments, *CapI-pb* expression is most pronounced in T5–T7. Expression in the VNC is no longer detectable. On the dorsal side, expression extends to the dorsal midline only in T5–T7. *CapI-pb* is now also expressed in the lateral wall of the foregut (Figure 4F). Expression of *CapI-pb* is downregulated during late larval stages and is barely detectable by stage 9.

### Larval Expression of *CapI-Hox3*

**Figure 5:**
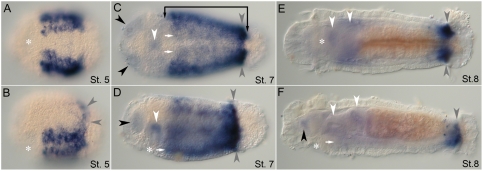
Larval expression of *CapI-Hox3*. Anterior is to the left for all panels. Stages indicated at bottom right of each panel. (A), (C), and (E) show ventral views; (B), (D), and (F), lateral views with ventral down. Position of the mouth is marked by an asterisk. (A, B) *CapI-Hox3* is expressed in the ventrolateral ectoderm of all segments and in the growth zone (stage 5) (gray arrowheads). (C, D) Expression in the lateral epidermis (bracket) and VNC (VNC, white arrows) of all segments, and in the posterior growth zone (gray arrowheads) at stage 7. Black arrowhead marks brain expression. White arrowhead marks expression in the dorsal wall of the stomodeum. (E, F) At late stage 8/early stage 9, expression is prominent in the posterior segments and growth zone (gray arrowheads), and weak in the VNC (white arrow), esophagus and pharynx (white arrowheads), and brain (black arrowhead).

Like the anterior class *Hox* genes, *CapI-Hox3* expression is initiated prior to segment formation (stage 4; unpublished data). The same pattern persists into stage 5, and includes expression in a large ventro–lateral ectodermal domain spanning all but the most anterior segment (Figure 5A and 5B). In addition, a band of expression in the posterior growth zone extends around the larva circumference (Figure 5B). Expression in the ventro–lateral ectoderm is somewhat discontinuous, and reflects belly plate formation at this stage. Over time, expression expands to include the ventral and lateral ectoderm of all larval segments (stage 5–7), and is most strongly expressed in the posterior growth zone (Figure 5C and 5D). Dorsal ectoderm expression is absent. During stage 7, two additional discrete expression domains appear in the brain and the distal portion of the stomodeum (Figure 5C and 5D). The stomodeal expression of *CapI-Hox3* is in a similar position to that of *CapI-lab*, but occupies a smaller area and is less prominent. During stages 8 and 9, strong expression persists in the posterior growth zone and the 2 posterior-most segments (Figure 5E and 5F). Weak expression of *CapI-Hox3* persists in the brain, VNC, pharynx, and esophagus.

### Larval Expression of the Central Class *Hox* Genes *CapI-Dfd*, *CapI-Scr*, *CapI-lox5*, *CapI-Antp*, *CapI-lox4*, *and CapI-lox2*

**Figure 6:**
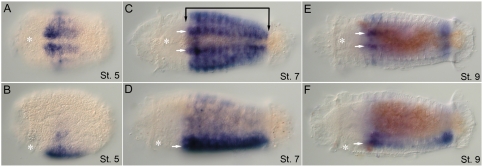
*CapI-Dfd* larval expression patterns. Anterior is to the left for all panels. Stages are written at the bottom right of each panel. (A), (C), and (E) show ventral views; (B), (D), and (F), lateral views. Asterisk marks position of the mouth. (A, B) At late stage 4, there is ventral ectodermal expression along the medial border of the belly plates. (C, D) At stage 7, expression includes the VNC (white arrows) and lateral ectoderm from T2 to the telotroch. (E, F) Expression is most prominent in the VNC of T2 (white arrows) and in the posterior-most segments and growth zone. Low levels of body epidermis expression persist.

In contrast to anterior class *Hox* genes and *Hox3* expression patterns, central class *Hox* gene expression is limited to the segmental ectoderm and growth zone. *CapI-Dfd* expression is first detected on both sides of the ventral midline along the medial border of the belly plates in the presumptive VNC at early stage 5 (Figure 6A and 6B). During segment formation and elongation of the larva, this expression domain expands posteriorly and laterally. At stage 7, *CapI-Dfd* broad ectodermal expression extends from the posterior half of T2 to the posterior growth zone (Figure 6C and 6D). There is prominent labeling in the VNC and ventro–lateral sides of the epidermis, and weaker expression in lateral and dorso–lateral areas. Expression is absent from the dorsal midline (Figure 6C and 6D). During stages 8 and 9, *CapI-Dfd* expression is downregulated. In stage 9 larvae, expression is strongest in T2 and the most posterior segments and growth zone. Lower levels of expression persist in between these two regions (Figure 6E and 6F).

**Figure 7:**
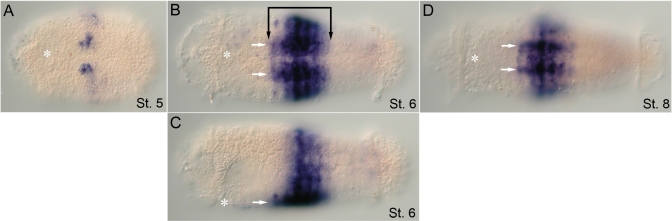
Expression patterns of *CapI-Scr* during larval stages. Anterior is to the left for all panels. Stages are indicated at bottom right of each panel. (A), (B), and (D) show ventral views; (C), lateral view. Asterisk marks position of the mouth. (A) Initiation of *CapI-Scr* expression at late stage 4 straddling the midline in T3 and T4 with weaker ventro-lateral expression in these segments. (B, C) Expression is in the VNC (white arrows) and lateral ectoderm in segments T3 to T7 (bracket). (D) Expression at stage 8 is very similar to the pattern at stage 6 (compare with [B]).

*CapI-Scr* expression is initiated as the first segments form in bilateral domains at the medial border of the belly plates in T3 and T4 at early stage 5, with weaker expression in the ventro–lateral ectoderm of these segments (Figure 7A). In mid-larval stages (stage 6), *CapI-Scr* is expressed in the VNC and ectoderm of T3–T7, most prominently in T5. Expression extends around the circumference of the larva, but is limited to T5 at the dorsal midline (Figure 7B and 7C). Very low levels of expression can be detected in T8 and the abdominal segments after an extended color development reaction (unpublished data). The same pattern observed at stage 6 persists to stage 8, although at lower levels (Figure 7D).

**Figure 8:**
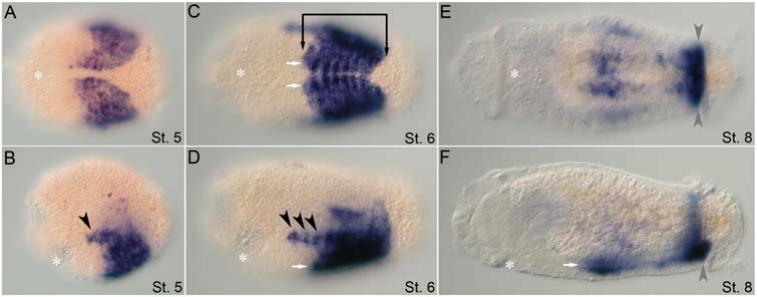
Larval expression of *CapI-lox5*. Anterior is to the left for all panels. Stages are indicated at bottom right of each panel. (A), (C), and (E) show ventral views; (B), (D), and (F), lateral views with ventral down. Mouth is marked by an asterisk. (A, B) Expression in the ventrolateral ectoderm extends from T4 to the posterior border of the belly plates at early stage 5. Lateral bands extend rostrally from the broad expression domain (black arrowhead). (C, D) Expression is in the VNC (white arrows), ventrolateral ectoderm, and in segmentally iterated lateral patches (black arrowheads). (E, F) At stage 8, *CapI-lox5* expression becomes most prominent in the VNC (white arrows) and posterior growth zone (gray arrowheads).

The *CapI-lox5* transcript is first detected at stage 5 in large ventro–lateral ectodermal domains, extending to the ventral and posterior borders of the belly plates on both sides of the ventral midline (Figure 8A and 8B). This expression pattern expands as additional segments form. A lateral band of expression extends anterior from the anterior face of the main expression domain (Figure 8B and 8D). At stage 6, *CapI-lox5* expression is mostly restricted to the ventro–lateral part of the segmental ectoderm with an anterior expression boundary in the VNC of T4 (Figure 8C). Laterally positioned patches of cells (one segment wide) in T2 and T3 are also present, giving the pattern a “wing-like” appearance (Figure 8D). Expression is downregulated in thoracic and anterior abdominal segments at early stage 8, with residual expression in the VNC and ventro–lateral epidermis. More prominent expression is detected in the ectoderm of the two posterior-most segments and in the growth zone (Figure 8E and 8F).

**Figure 9:**
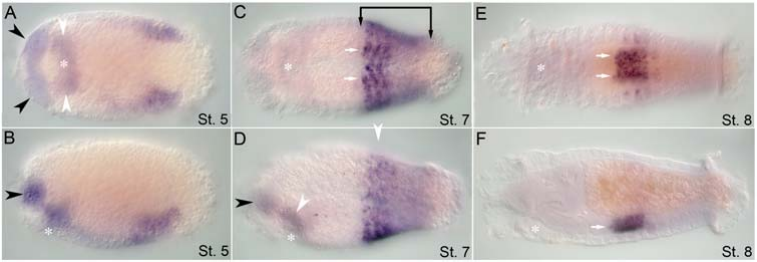
*CapI-Antp* expression during larval development. Anterior is to the left for all panels. Stages are at the bottom right of each panel. (A), (C), and (E) show ventral views; (B), (D), and (F), lateral views with ventral down. Asterisk marks the position of the mouth. (A, B) *CapI-Antp* is expressed in the posterior half of the mid-body in the ventrolateral ectoderm, the brain (black arrowhead), and the presumptive foregut (white arrowheads). (C, D) Expression in the VNC (white arrows) and lateral ectoderm spans segments T6 to the telotroch (bracket). Expression is consistently strongest in T6 to T8. Weak expression in brain (black arrowhead) and foregut (white arrowhead) is still detectable. (E, F) Expression is largely restricted to the VNC of thoracic segments T5 to T9 by stage 8 (white arrows).

*CapI-Antp* expression is first detectable in the bilobed brain and presumptive foregut at stage 5 (Figure 9A and 9B). Expression in the ventro–lateral ectoderm of the posterior half of the trunk appears soon thereafter, and by stage 7, it has expanded circumferentially to span the region from T6 to the telotroch (Figure 9C and 9D). Expression is strongest in the four anterior-most segments of this domain. Weak expression of *CapI-Antp* persists in the brain and foregut. In the transition to stage 8, a posterior expression boundary appears, and prominent expression becomes limited to the VNC of segments T5–T8 (Figure 9E and 9F). The anterior expression border is in the posterior side of T5. Weaker expression is visible in T9 and lateral ectodermal cells of T5–T8.

**Figure 10:**
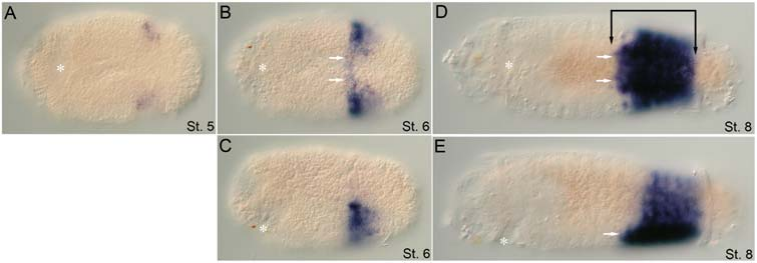
Larval expression of *CapI-lox4*. Anterior is to the left for all panels. Stages indicated at bottom right of each panel. (A), (B), and (D) show ventral views; (C) and (E), lateral views with ventral down. Asterisk marks position of the mouth. (A, B) Onset of *CapI-lox4* expression at early stage 5 in a ventro-lateral ectodermal domain in the posterior mid-body. (C, D) By stage 6, expression crosses the midline in T7 and connects the two ventro-lateral domains. (E, F) *CapI-lox4* expression is most prominent at stage 8 and includes T7 to T9, all of the abdominal segments, and the growth zone (black bracket). White arrows point to VNC expression (white arrows).

*CapI-lox4* expression initially appears as a small domain of ventro–lateral ectodermal cells in the posterior quarter of the mid-body at stage 5 (Figure 10A). In contrast to most other *Capitella Hox* genes, expression is initially absent from the ventral midline and VNC. At stage 6, expression is predominantly in T7–T9, with decreasing levels from anterior to posterior (Figure 10B and 10C). In T7, new expression extends across the ventral midline, connecting the lateral expression domains. During stage 7, expression expands posteriorly, across the ventral midline, and laterally/dorsally. At stage 8, *CapI-lox4* is strongly expressed in the VNC and segmental ectoderm of T7–T9, all abdominal segments, and the posterior growth zone (Figure 10D and 10E). Expression also expands to the dorsal side of the body (Figure 10E).

**Figure 11:**
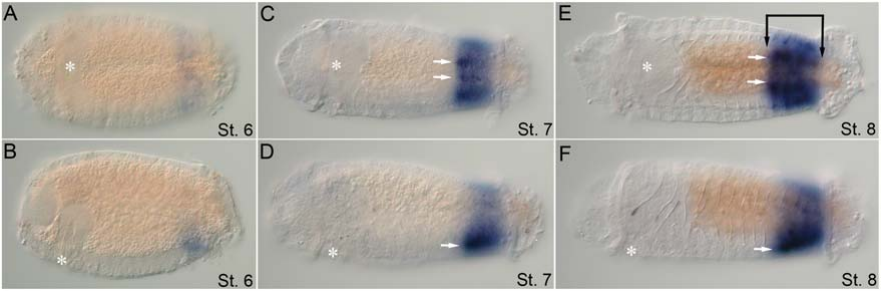
Larval expression of *CapI-lox2*. Anterior is to the left for all panels. Stages are written at the bottom right of each panel. (A), (C), and (E) show ventral views; (B), (D), and (F), lateral views with ventral down. The position of the mouth is marked by an asterisk. (A, B) *CapI-lox2* expression is in the ventral and ventrolateral part of the ectoderm of the abdominal segments during stage 6. (C, D) By stage 7, expression in the VNC (white arrows) and ventral and lateral epidermis extends from A1 to the growth zone. (E, F) *CapI-lox2* expression at stage 8 includes ectodermal expression in A1 to A4 and the growth zone (black bracket).

*CapI-lox2* expression is initiated slightly after *CapI-lox4* during stage 5; however, detection is possible only after a long staining reaction (not shown). At stage 6, weak expression of *CapI-lox2* is detectable in the ventro–lateral ectoderm of the abdominal segments (Figure 11A and 11B). By stage 7, strong expression in the VNC and ventral and lateral ectoderm extends from A1 to the growth zone (Figure 11C and 11D). Over time, expression expands into newly formed segments, including all abdominal segments formed during larval stages (A1–A4; Figure 11E and 11F). *CapI-lox2* expression is absent from the dorsal midline.

### Larval Expression of the Posterior Class *Hox* Genes *CapI-Post1* and *CapI-Post2*

*CapI-Post1* expression was not detectable in broad ectodermal expression domains of the larva using any of three different probes, although expression was observed in the chaetal sacs of developing chaetae (unpublished data).

**Figure 12:**
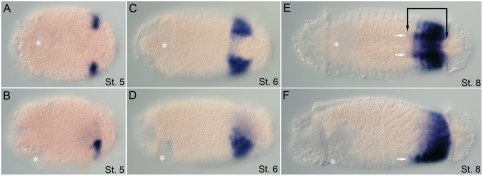
*CapI-Post2* expression in larval stages. Anterior is to the left for all panels. Stages are marked at the bottom right of each panel. (A), (C), and (E) show ventral views; (B), (D), and (F), lateral views with ventral down. The mouth is marked by an asterisk. (A, B) Initial expression is in ventrolateral ectodermal bands that span segments A1 and A2 during stage 5. Note absence of expression across the ventral midline. (C, D) By stage 6, additional abdominal segments have formed, and these segments also express *CapI-Post2*. Expression across the ventral midline appears, although it is weaker than the lateral expression domains. (E, F) At stage 8, expression is apparent in the VNC (white arrows) and in the ventral and lateral epidermis of all abdominal segments (black bracket).

*CapI-Post2* expression is initiated at the same time as *CapI-lox2* (stage 5), although at higher levels. Expression is in bilateral ectodermal bands of the two abdominal segments present at this stage (A1 and A2), confined to the ventro–lateral region of these segments, and absent from the ventral midline (Figure 12A and 12B). As the larva elongates and additional segments form, expression expands posteriorly (Figure 12C and 12D). During stage 6 in the anterior-most abdominal segments, expression now spans the ventral midline, connecting the lateral expression domains (Figure 12C). Expression gradually expands dorsally but does not connect at the dorsal midline (Figure 12D). At stage 8, *CapI-Post2* is expressed in the VNC and lateral ectoderm of all abdominal segments, with an anterior boundary of A1 (Figure 12E and 12F). In A1, expression is generally limited to the VNC.

### *Capitella Hox* Gene Expression in Juveniles

**Figure 13:**
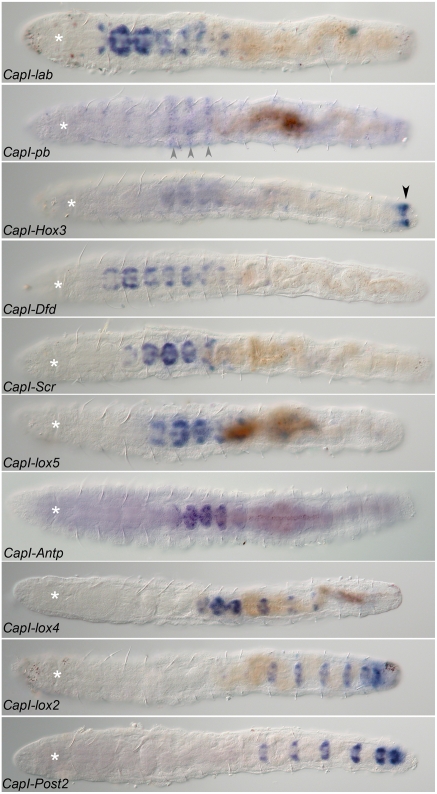
*Hox* gene expression in juveniles. All panels are ventral views with anterior to the left. Asterisk marks the position of the mouth. Gene names are in the lower left corner of each panel. Age of animals shown is 3 d after metamorphosis. *Capitella Hox* gene expressions are largely limited to a subset of VNC ganglia in juveniles. Exceptions include *CapI-Hox3*, which is expressed in the prepygidial growth zone (black arrowhead), and *CapI-pb*, which shows weak epidermal stripes (gray arrowheads).

In contrast to the broad ectodermal patterns expressed during larval development, *Capitella Hox* gene expression in juveniles is generally restricted to the VNC (Figure 13), with a few notable exceptions (see below). *Hox* gene expression in juveniles exhibits precise anterior and posterior boundaries, and expression is limited to 4 to 7 segments (Figure 13). In juveniles, the 2 to 3 anterior-most ganglia are out of register with segmental boundaries, and straddle adjacent segments. We report expression as it corresponds to segmental boundaries. The anterior class *Hox* gene *CapI-lab* is expressed from the anterior side of T2 (first ganglion) to T8. *CapI-Dfd* shows expression from the posterior side of T2 (second ganglion) to T8. *CapI-Hox3* is prominently expressed in the posterior growth zone, and is also detectable in T2 through T8 (weaker in T2 and T3). The anterior central class genes *CapI-Scr* and *CapI-lox5* are expressed in segments T3 to T8 and T4 to T8, respectively. *CapI-Antp* expression is in segments T5 to T9, and *CapI-lox4* is expressed in T7 to T9 and the anterior abdominal segments, A1 to A3. The expression patterns of *CapI-lox2* and *CapI-Post2* appear identical; VNC expression is observed in all abdominal segments. Newly formed ganglia exhibit the strongest expression of *CapI-lox2* and *CapI-Post2*. *CapI-Post1* expression is not detectable in juveniles. The juvenile expression patterns contrast with larval patterns for *CapI-lab*, *CapI-pb*, *CapI-Hox3*, *CapI-Dfd*, *CapI-lox5*, and *CapI-Antp*, which are initially broadly expressed and share the same posterior boundary (the posterior growth zone). At late larval stages (stages 8/9), these patterns have been refined, and in most cases they predict juvenile anterior and posterior expression boundaries.

In juveniles, only *CapI-lab*, *CapI-pb*, and *CapI-Hox3* are expressed outside the VNC. Esophageal *CapI-lab* expression is limited to the posterior portion of the esophagus. These *CapI-lab–*expressing cells have a neural-like morphology, and are likely a subset of the stomatogastric nervous system or sensory cells connecting to the esophagus. The segmental epidermal stripe pattern of *CapI-pb* observed in late larval stages persists into juvenile stages, albeit at lower levels, with the most discrete stripes in T5 to T7. There is also expression in the prepygidial epidermis. *CapI-Hox3* is expressed prominently in the mesoderm of the posterior growth zone, and is the only *CapI-Hox* gene expressed in the posterior growth zone of juveniles.

## Discussion

### *Capitella* sp. I *Hox* Cluster and Evolution of the Lophotrochozoan *Hox* Cluster

*Hox* genes form a class of highly conserved genes that play key roles in body plan regionalization. In addition, in a number of cases for which genomic information is available, *Hox* genes appear in clusters, presumably reflecting their evolutionary origin by tandem duplication. Although there are several studies of *Hox* genes for various annelids (e.g., *Chaetopterus* [10], *Nereis virens* [11], and *Helobdella triserealis* [12]) and other lophotrochozoans such as molluscs [17], [18], [46], nemerteans [23], platyhelminthes [19]–[21], [35], and brachiopods and priapulids [37], the linkage of *Hox* genes in the genome of *Capitella* sp. I is the first direct evidence of a *Hox* cluster in the Lophotrochozoa. Taken together, the presence of a *Hox* cluster in Deuterostomia [47]–[53], Ecdysozoa [54]–[56], and our study representing the Lophotrochozoa provides compelling support for the interpretation that the protostome/deuterostome ancestor also possessed a *Hox* gene cluster.

Eight of the 11 *Capitella* sp. I *Hox* genes are genomically linked within a 243-kb region, and two additional *Hox* genes are linked on a separate contig (*CapI-lox2* and *CapI-Post2*), spanning 21.6 kb. Only *CapI-Post1* is not clustered with any other *Capitella Hox* genes. Therefore, we cannot directly demonstrate the presence of a single intact cluster containing all *Capitella Hox* genes. If additional evidence demonstrates genome linkage between the two contigs containing multiple *Hox* genes, this cluster would span at least 345 kb, larger than vertebrate *Hox* clusters (70 kb to 180 kb) [57], but much smaller than some intact arthropod *Hox* clusters (*S. gregaria*, 700 kb–2 Mbp [58]; *T. castaneum*, 756 kb [59]). All clustered *Capitella* sp. I *Hox* genes are transcribed in the same direction. Furthermore, no additional predicted genes are identified between adjacent *Hox* genes, characteristic of *Hox* clusters in chordates and *Tribolium castaneum* [59]. In contrast, the ANT-C cluster of *Drosophila* contains multiple genes between *Hox* genes, and the transcriptional orientation of *Dfd* and *ftz* is reversed with respect to the order and orientation of other genes within the complex [7].

The size of *Hox* transcription units varies greatly among metazoans. Most *Capitella* sp. I transcription units are predicted to be 4.0–6.2 kb, although *CapI-Dfd* is smaller (1.5 kb), and *CapI-pb* and *CapI-Hox3* are larger (9.2 kb and 27.8 kb, respectively). In contrast, *Drosophila Hox* genes range from 6–70 kb, and *Antp* is 103 kb [7]. Vertebrate transcription units are much smaller [60], with the first 9 genes of the human *HoxA*-cluster ranging from 2.25 kb to 3 kb, with the exception of *HoxA3* (20.8 kb).

We also examined the genomic organization of the *Capitella* sp. I *Parahox* genes, whose expression we previously described [27]. *CapI-Xlox* and *CapI-Cdx* (transcription unit size of 7 kb and 6.35 kb. respectively) are located approximately 33 kb apart on the same contig (scaffold 444), whereas *CapI-Gsx* is on a separate contig (scaffold 760). In contrast to *Capitella Hox* genes, *CapI-Xlox* and *CapI-Cdx* have opposite transcriptional orientations. In addition, there is at least one predicted gene between them, and several genes flank *CapI-Xlox* and *CapI-Cdx*. None of the *Parahox* genes are linked to any contigs containing *Hox* genes. In vertebrates, the homeodomain-containing genes *eve* and *mox* are linked to the *Hox* genes, a genomic organization known as the extended *Hox* cluster [61]. *Capitella* sp. I orthologs of *eve* and *mox* are not linked to any of the *Capitella Hox* genes (Fröbius and Seaver, unpublished data).

Our identification and analyses of 11 *Capitella* sp. I *Hox* genes, including 6 central class members, advances our understanding of protostome *Hox* cluster evolution. It has been suggested that the ancestral protostome *Hox* cluster contained between 8 and 11 genes [37]. The imprecision in this number reflects uncertainty in the timing of specific paralog group duplication events within the Ecdysozoa and Lophotrochozoa [24]. Although the presence of a single member of PG1–PG5 in the ancestral protostome *Hox* cluster is strongly supported, the evolution of the other central and posterior class *Hox* genes is less clear. The identification of a definitive PG7 gene (*CapI-Antp*) that clusters with several other lophotrochozoan genes, ecdysozoan *Antp*, and a chaetognath PG7 gene (*Fen Hox7*), and its genomic position between *Lox5* and *Lox4* in the *Capitella Hox* cluster, strongly suggests that a single PG7 class gene was present in the ancestral protostome cluster. The hypothesis that ecdysozoan *ftz* and lophotrochozoan *Lox5* genes are PG6 orthologs, and not the result of clade-specific duplication events [62], is also supported by our analyses. From our analyses and those of others, there are currently insufficient data to determine the relationship among PG8 genes (*Lox4/Lox2/Ubx/AbdA*). *Lox4/Lox2* genes do not form a monophyletic clade in our analyses, although *Ubx/AbdA* genes do, suggesting that *Lox4* and *Lox2* as well as *Ubx* and *AbdA* arose by separate duplication events in lophotrochozoans and ecdysozoans, respectively [32], [37]. With additional sampling in the Ecdysozoa and Lophotrochozoa, we will likely be able to determine the timing of these paralog group duplications. Posterior *Hox* genes appear to be especially labile (“posterior flexibility” [52]), and likely have independently duplicated in the three bilaterian clades, making it difficult to determine paralogy. The protostome ancestor likely possessed a *Hox* cluster of 9–11 genes, including two anterior class genes (*Labial* and *pb*), a single *Hox*3 gene, 5 to 6 central class genes (*Dfd*, *Scr*, *Lox5/ftz*, *Antp*, and *Lox4/Lox2/Ubx/AbdA*), and 1 to 2 posterior genes. It is noteworthy that the same 11 *Hox* genes have also been reported for the polychaete annelid *N. virens* [11]. The 11 *Hox* genes of *Capitella* sp. I that are arranged into 1 to 2 “organized clusters” [3] in the genome (except *CapI-Post1*) share the same transcriptional orientation, lack non-*Hox* genes interspersed among them, and appear to approximate the prototypical and ancestral organization of the protostome-deuterostome *Hox* cluster.

### Temporal and Spatial Colinearity of *Capitella Hox* Gene Expression

**Figure 14:**
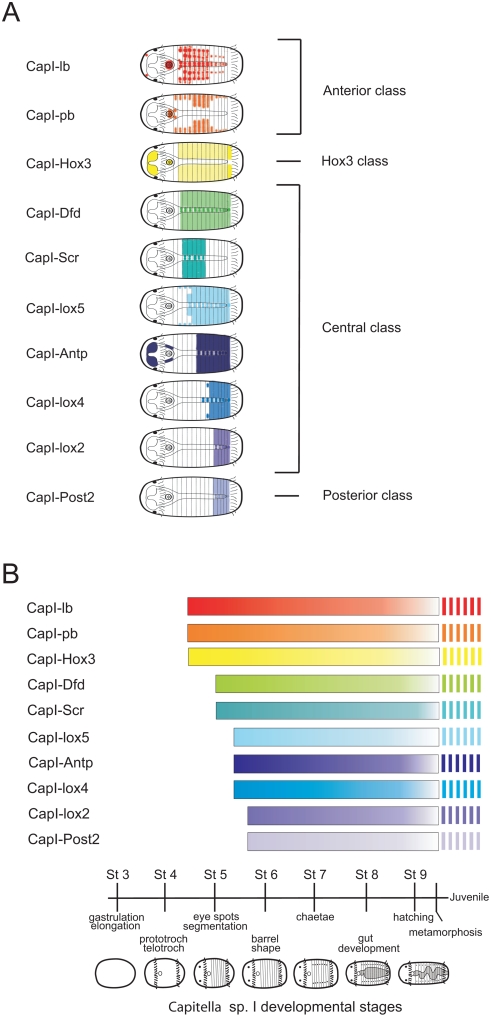
Summary of *Capitella* sp. I spatial and temporal *Hox* gene expression in larvae. (A) Schematic of larval expression patterns of 10 *Hox* genes. Patterns illustrated are for a mid-larval stage. Larvae have 13 segments at stage shown. (B) Diagram displaying the temporal onset of expression for the genes displayed in (A). Vertical stripes indicate persistence of expression into juvenile stages. Schematic of *Capitella* sp. I ontogenesis following gastrulation is shown at bottom of figure.

All 10 clustered *Capitella* sp. I *Hox* genes display unique expression patterns, and their expression is initiated within a narrow time frame during larval development, which can be clearly distinguished into four temporal classes (Figure 14B). The earliest genes to initiate expression are the anterior class *Hox* genes *CapI-lab* and *CapI-pb*, and *CapI-Hox3*, which occur before the morphological appearance of segments. *CapI-Dfd* and *CapI-Scr* expression is initiated shortly afterwards as the first segments appear, followed by *CapI-lox5*, *CapI-Antp*, and *CapI-lox4* expression. The latest *Hox* genes to initiate expression are *CapI-lox2* and *CapI-Post2*. Each *Capitella* sp. I *Hox* gene exhibit its broadest and highest expression level at a unique stage, reflecting the order of activation for each gene. Following this peak of expression, *Hox* genes are generally down-regulated, and only weak expression levels are detectable by the end of larval development. The temporal sequence of *Hox* gene activation in *Capitella* sp. I is correlated with the sequence of these genes in the genomic cluster, characteristic of temporal colinearity. In *Chaetopterus*, the expression of *Hox1/lab*, *Hox2/pb*, *Hox3*, *Hox4/Dfd*, and *Hox5/Scr* [10] exhibits staggered temporal onset of expression. Presuming a genomic organization similar to that observed in *Capitella*, these genes would fit a temporal colinearity paradigm. In contrast, the onset of *Hox* gene expression in *Nereis virens* [11], *Platynereis dumerilii*, and the four *Hox* genes characterized in *Helobdella* (*lb*, *Dfd*, *Scr*, and *Antp* orthologs [12]) does not fit a possible temporal colinearity scenerio.

**Figure 15:**
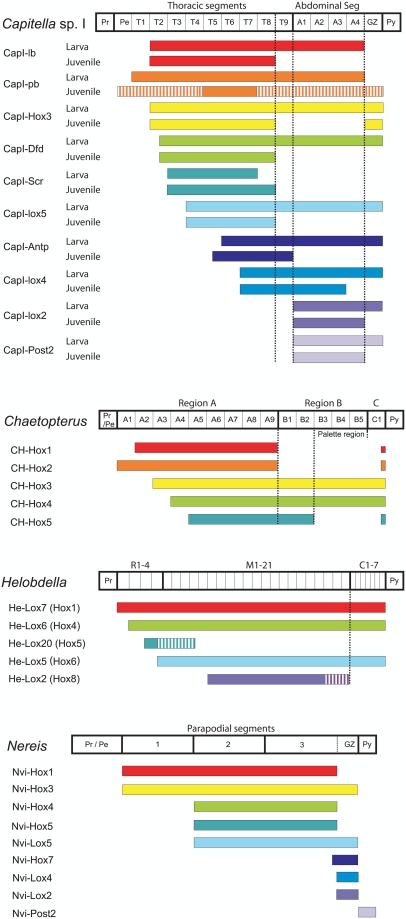
Comparison of *Hox* gene expression patterns across annelids. Generalized diagram comparing boundaries of *Hox* gene expression along the main body axis of *Capitella* sp. I with expression boundaries in *Helobdella*, *Chaetopterus*, and *Nereis*. Body axis schematics are shown as boxed diagrams next to species names and include segments and tagmata for each species. Solid bars indicate strong expression and striped bars indicate weaker expression. For *Nereis*, the anterior border shown is the anterior *Hox* gene boundary at the earliest stage expressed. Pr indicates prostomium; Pe, peristome; and GZ, growth zone. Taxon-specific abbreviations: *Capitella*: T, thoracic segments; A, abdominal segments; and seg, segments. *Chaetopterus*: A, B, C, segments of body region A, B, and C, respectively. *Helobdella*: R, rostral segments; M, medial segments; and C, caudal segments.

During larval development, *Capitella* sp. I *Hox* genes are broadly expressed in the ectoderm, which is most prominent in (and in some cases restricted to) the segmental portion of the body. The anterior-most *Hox* gene expression boundary is that of *CapI-pb*, whose anterior boundary is immediately posterior to the mouth. In both larval and juvenile stages, anterior boundaries of adjacent *Hox* genes are staggered (Figures 14A and 15), displaced by one or two segments from that of the adjacent gene, which is consistent with a role in influencing the identity of one or two adjacent segments. Following the rule of spatial colinearity, anterior expression borders are generally arranged in the same order from anterior to posterior as their 3′ to 5′ genomic position in the cluster. With the exception of T5, each of the nine thoracic segments has a unique *Hox* expression boundary (either anterior or posterior boundary). In the abdomen, only A1 has a unique *Hox* boundary. *CapI-pb* expression is distinct from the contiguous ectodermal expression domains of other *Hox* genes; it has a stripe pattern in all segments, suggesting involvement a process other than anterior-posterior patterning. None of the *Capitella* sp. I *Hox* genes is expressed in the unsegmented posterior terminus, in contrast with pygidial expression of several *Hox* genes in *Nereis* and *Platynereis* [11]. Our results demonstrate temporal and spatial colinearity of *Hox* gene expression in a lophotrochozoan, and are consistent with an ancestral role for *Hox* genes in patterning the antero-posterior axis of the epidermis and central nervous system. The presence of spatial and temporal colinearity in all three bilaterian superclades indicates these features were likely present in the protostome–deuterostome ancestor.

Three exceptions to the rule of spatial colinearity are observed in *Capitella* sp. I: the anterior boundary of *CapI-pb* is displaced anterior to that of *CapI-lb*, *CapI-Hox3*, and *CapI-lab* have the same anterior expression boundary, and *CapI-lox2* and *CapI-Post2* share the same anterior and posterior boundaries. Both *CapI-pb* and *CapI-Hox3* also exhibit noncanonical *Hox* gene expression (see below). The anterior shift of the *Hox2/pb* expression boundary relative to that of *Hox1/lab* is widely found across taxa, including vertebrate examples such as mouse and zebrafish [63], and the polychaetes *Chaetopterus* and *Platynereis*. Therefore, this shift may represent a general feature rather than an exception [10], [11], although arthropods do not appear to show an anterior shift of *pb* relative to *lab*, and *Drosophila pb* is displaced caudally [64]. *Hox1* and *Hox3* orthologs also share an anterior expression boundary in *Nereis virens* and the spider *Cupiennius salei* [65]. Leech *Hox2/pb* and *Hox3* orthologs have not been isolated, and we were unable to identify them in searches of the *Helobdella* genome (http://genome.jgi-psf.org/Helro1/Helro1.home.html). Since there is a gap of only a single segment between the anterior boundaries of *He-Lox7* (*Hox*1) and *He-Lox6* (*Hox*4), there may be a loss of *Hox2* and *Hox3* paralogs in the leech genome [12]. Although *CapI-lox2* and *CapI-Post2* share the same anterior and posterior expression boundaries and are adjacent to one another in the genome, their expression patterns show gene-specific characteristics. *CapI-lox2* is expressed across the ventral midline and in the VNC, where *CapI-Post2* is absent, and the two genes show varying expression levels at their anterior boundary.

One of the striking findings from our study is the biphasic nature of *Hox* gene expression patterns between larval and juvenile stages in *Capitella* sp. I. During larval stages, most *Hox* genes are broadly expressed in the ectoderm and developing VNC, largely share a common posterior boundary at the posterior growth zone, and have gene-specific anterior expression boundaries. In the transition from larval to juvenile stages, two features of *Hox* gene expression change: almost all expression becomes limited to the VNC, and discrete posterior expression boundaries appear. Gene-specific larval anterior expression boundaries are maintained into juvenile stages for all *Hox* genes except for *CapI-Antp*, in which the anterior boundary shifts rostrally by one half-segment. In juveniles, the expression of each *Hox* gene spans a few segments, and adjacent *Hox* genes show partially overlapping but staggered domains of expression in the VNC. For *CapI-lox2* and *CapI-Post2*, newly formed posterior ganglia exhibit the strongest expression, reminiscent of the slight gradient seen in *Nereis virens* for these two genes [11]. Since *Capitella* sp. I continues to add segments throughout its life, and undergoes robust posterior regeneration from multiple axial positions, there may be a need to maintain axial information into adulthood. Distinct expression patterns between larval and juvenile stages have not been reported in other annelids. *Chaetopterus Hox* genes are expressed in the VNC during larval development, although expression in juveniles has not been described [10]. In *Nereis*, *Hox1*, *Hox4*, *Hox5*, and *Lox5* expression later become more restricted to the VNC in nectochaetes, but nested central nervous system (CNS) expression does not persist into juvenile stages [11].

During larval stages, nearly all *Capitella* sp. I *Hox* genes are expressed in the posterior growth zone, a region that generates larval segments 10–13, and all segments formed after metamorphosis. As expression patterns mature during late larval and early juvenile stages, posterior expression boundaries appear anterior to the growth zone. Only *CapI-Hox3* shows persistent growth zone expression in juvenile stages. This situation contrasts with a greater number of *Hox* genes in *Nereis* juveniles that exhibit posterior growth zone expression, including *lox2*, *post2*, *Hox3*, *Lox5*, *Hox7* (*Antp*), and *Lox4*. During early larval stages in *Chaetopterus*, transient growth zone expression is observed for *CH-Hox1*, *CH-Hox2*, *CH-Hox3*, *CH-Hox4*, and *CH-Hox5* [10].

### Correlation of *Hox* Expression with Morphological Boundaries

Expression boundaries of several *Hox* genes correlate with the transition between the thorax and abdomen in *Capitella* sp. I (Figure 15). In larvae and juveniles, the anterior expression boundaries of *CapI-lox2* and *CapI-Post2* mark the thoracic–abdominal boundary. After metamorphosis, when distinct posterior expression boundaries appear, *CapI-Antp* also has a posterior expression boundary at the thoracic–abdominal junction. *CapI-lb*, *Hox3*, *CapI-Dfd*, *CapI-Scr*, and *CapI-lox5* all have posterior boundaries at the anterior edge of T9 in juveniles, although each gene has a distinct anterior boundary. *CapI-lox4* is the only *Hox* gene expressed in both thoracic and abdominal tagma, and whose expression spans the thoracic–abdominal boundary. It is striking that expression boundaries for 8 of the 10 *Hox* genes coincide with either the anterior or posterior side of segment T9, and suggests that the division between the thorax and abdomen in *Capitella* sp. I is a transition zone the width of one segment, rather than a narrow boundary. The segment T9 has a mix of both thoracic and abdominal characteristics. The thoracic ganglia are closely spaced relative to the substantially greater distances between ganglia of the abdomen; the T9 ganglion shows spacing typical for thoracic ganglia. However, T9 has hooded hook chaetae, a characteristic of abdominal segments [66]. Organization of the VNC connectives is notably different between the thorax and abdomen (unpublished data), and T9 shows thoracic-like charactistics on the anterior face of its ganglion and more distinct and widely spaced connectives on its posterior face, typical of the abdominal ganglia.

A single posterior boundary shared by multiple *Hox* genes that correlates with a major body transition is also observed in other animals. In *Chaetopterus*, posterior boundaries of *CH-Hox1* and *CH-Hox2* expression coincide with the boundary between tagma A and B, and the posterior boundary of *CH-Hox5* marks the anterior boundary of the palette segments in tagma B (Figure 15) [10]. In leeches, the posterior boundary of *lox2* and *lox4* is at the anterior edge of the caudal ganglion, a body transition (Figure 15) [12]–[14], [16]. In spiders, five of the anterior *Hox* genes share a common posterior boundary between the prosoma and opistosoma [67].

### Noncanonical Expression of *Capitella* sp. I *Hox* Genes

Although all *Capitella* sp. I *Hox* genes except *Post1* (see below) show nested sets of trunk ectodermal and neuroectodermal expression, *CapI-lab*, *CapI-pb*, *CapI-Hox3*, and *CapI-Antp* have additional expression domains, some of which are conserved with other taxa. Both *Capitella* and *Platynereis* have *Hox1/lab*-positive cells in the head epidermis, although *Nereis* does not [11]. These *Platynereis Hox1/lab*-positive cells are apical tuft cells, a cell type that neither *Nereis* nor *Capitella* have. *Hox1/lab* expression is generally restricted to post-oral regions in other animals. *CapI-Hox3* and *CapI-Antp* are both expressed in the brain. To our knowledge, expression of *Hox3* and *Antp* orthologs or other *Hox* genes in the brain has not been reported for other protostomes.

*CapI-lab*, *CapI-pb*, *CapI-Hox3*, and *CapI-Antp* are expressed in the presumptive foregut. Foregut expression of *Hox1/lab* and *Hox2/pb* orthologs is also reported for *Chaetopterus* [10], and *Hox1* is expressed at the foregut–midgut boundary in *Nereis* and *Platynereis* metatrochophores. To our knowledge, foregut expression of *Hox3* and *Antp/Hox7* has not been reported for annelids other than *Capitella*. Outside annelids, anterior *Hox* gene expression in the developing digestive tract is observed in hemichordates [68], chordates including *Branchiostoma* [69], and *Drosophila* [64]. Although foregut tissues originate from different germ layers in distinct taxa, early expression of anterior class *Hox* genes associated with the foregut among deuterostomes, lophotrochozoans, and ecdysozoans appears to be conserved.

Expression data for *Post1* in lophotrochozoans are quite limited. In the cephalopod *Euprymna scolopes*, *Post1* is expressed in the developing ganglia of the brachial crown, but direct comparison with annelids is difficult due to the specialized cephalopod body plan [17]. Within annelids, *Post1* expression has only been reported for *N. virens* and *P. dumerilii*, and is detected in larval chaetal sacs in both polychaetes [11]. In *Capitella* sp. I, *CapI-Post1* is not clustered with any other *Capitella Hox* genes or expressed in broad ectodermal domains at any stage we examined, although we observed expression in the chaetal sacs. *CapI-Post1* may also be expressed during life history stages other than those we analyzed, is likely not involved in vectorial patterning, and may have been recruited for other functions.

### Hox Genes and the Evolution of the Annelid Larval Body Plan

Expression studies support the hypothesis that ancestral annelids used *Hox* genes in vectoral patterning along their main body axis. Due to its key role in axial patterning and segment identity, it is thought that *Hox* cluster evolution is intricately linked with the evolution of the body plan itself. The five annelids in which *Hox* gene expression has been studied have a number of important species-specific differences relevant to axial patterning. These differences include direct versus indirect development, differences in segment number generated during larval and adult stages, heteronomy versus homonomy, and morphology of the adult body plan. *Chaetopterus* exhibits the most complex body regionalization, with three distinct body regions and a number of segments that possess unique specialized structures [10]. By contrast, segments in *Nereis* and *Platynereis* are highly uniform, and distinct body regions are not morphologically distinguishable [70]. The distinct tagma of *Capitella* and *Helobdella* represent an intermediate level of body regionalization.

These diverse annelid body plans correlate with striking differences in *Hox* gene expression patterns. Anterior boundaries of orthologous *Hox* genes are not consistently expressed in the same segments across species (Figure 15). Thus, the use of molecular criteria to assign homologous segment identity among annelids may not be possible. In contrast, a common theme across annelids is the apparent correlation between the presence of distinct body tagma and the presence of posterior *Hox* gene expression boundaries. Posterior expression boundaries in *Chaetopterus* correlate with the boundary between regions A and B, posterior expression boundaries correlate with the thoracic–abdominal transition in *Capitella*, and posterior expression boundaries correspond with the anterior boundary of the caudal ganglion in the leech. *Nereis* and *Platynereis* lack posterior *Hox* gene expression boundaries, consistent an absence of morphological boundaries. Thus, as previously proposed by Irvine [39], the presence of posterior boundaries of *Hox* gene expression in annelids correlates with species-specific body regionalization. If one assumes that annelids generally exhibit genomic linkage of *Hox* genes in the order found in *Capitella*, the five *Hox* genes of *Chaetopterus* and the clustered *Hox* genes of *Capitella* exhibit temporal colinearity, however, neither *Nereis* nor *Helobdella* do [11], [12]. Furthermore, *Nereis* and *Platynereis* lack spatial colinearity of anterior expression boundaries, and only two larval segments have corresponding *Hox* gene expression boundaries (Figure 15, [11]). *Nvi-Hox1* and *Nvi-Hox3* both have an anterior boundary at the first setiger, and *Nvi-Hox4*, *Nvi-Hox5*, and *Nvi-lox5* share an anterior boundary at the second setiger. *Nvi-Hox7*, *Nvi-Lox4*, and *Nvi-Lox2* are localized to the posterior growth zone, and *Nvi-Post2* is in the terminal pygidium. Even the direct developing leech exhibits staggered *Hox* expression boundaries, although onset of expression is at late stages and the genomic organization has yet to be reported [12], [13]. If the protostome/deuterostome ancestor exhibited spatial and temporal colinearity, nereid *Hox* gene expression appears to have relaxed regulation of expression, perhaps reflecting its highly homonomous body plan that lacks unique segmental identities. The variation in *Hox* gene expression among *Capitella* sp. I, *Helobdella*, *Platynereis*, *Nereis*, and *Chaetopterus* emphasizes the importance of comparative studies within a phylum. Investigations of species with different life histories and body plans play an important role in revealing insights into how *Hox* genes contribute to animal body plan evolution.

## Supporting Information

Figure S1 Nexus alignment used in phylogenetic analyses. A 72-amino acid alignment was constructed using the 60 amino acids of the homeodomain and the 12 amino acids immediately 3′ of the homeodomain from representative bilaterian taxa representing all of the Hox and Parahox PGs. Cc indicates *Capitella* sp. I; Nv, *Nereis virens*; Es, *Euprymna scolopes*; Tc, *Tribolium castaneum*; Bf, *Branchiostoma floridae*; Nw, *Nematoderma westbladi*; Sr, *Symsagitiffera roscofensis*; Fen, *Flaccisagitta enflata*; Sc, *Spadella cephaloptera*; Ls, *Lineus sanguineus*; Dj, *Dugesia japonica*; and Lin, *Lingula anatina*.
(0.02 MB PDF)

Figure S2 Neighbor-joining bootstrap consensus tree. A neighbor-joining (NJ) bootstrap consensus tree (using mean amino acid distances) was constructed using PAUP* v4.0b10 [29] with 1,000 iterations, using a 72-AA alignment of representative bilaterian Hox and Parahox genes (see Figure S1), including the 60-AA homeodomain as well as the 12 AAs immeditately flanking the 3′ end of the homeodomain. Numbers above branches indicate NJ bootstrap support, shown as a percentage. New *Capitella* sp. I sequences are shown in bold; all *Capitella* sequences are delimited by an arrow.
(0.39 MB TIF)

Figure S3 Maximum likelihood bootstrap consensus tree. A maximum likelihood (ML) bootstrap consensus tree was constructed using RAXML v2.2.1 [31] using the rtrev+G model of protein evolution, selected via ProtTest [30]. An initial search of 500 iterations was conducted to determine consistency of recovering the most likely tree (unpublished data). An additional 1,000 bootstrap iterations were conducted in RAXML v2.2.1. Numbers above branches indicate ML bootstrap support shown as a percentage. New *Capitella* sp. I sequences are shown in bold; all *Capitella* sequences are delimited by an arrow.
(0.40 MB TIF)
