# Supplementary material for: Genomic Organization and Expression Demonstrate Spatial and Temporal Hox Gene Colinearity in the Lophotrochozoan Capitella sp. I
Source: PLoS One. 2008 Dec 23;3(12):e4004. doi: 10.1371/journal.pone.0004004 (PMC2603591; doi:10.1371/journal.pone.0004004)
Supplement: Figure S1 — Nexus alignment used in phylogenetic analyses. A 72-amino acid alignment was constructed using the 60 amino acids of the homeodomain and the 12 amino acids immediately 3′ of the homeodomain from representative bilaterian taxa representing all of the Hox and Parahox PGs. Cc indicates Capitella sp. I; Nv, Nereis virens; Es, Euprymna scolopes; Tc, Tribolium castaneum; Bf, Branchiostoma floridae; Nw, Nematoderma westbladi; Sr, Symsagitiffera roscofensis; Fen, Flaccisagitta enflata; Sc, Spadella cephaloptera; Ls, Lineus sanguineus; Dj, Dugesia japonica; and Lin, Lingula anatina. (0.02 MB PDF) [file pone.0004004.s001.pdf]

```
#NEXUS
[!Created by MacVector on Apr 01 2007 at 18:26:53.]

BEGIN DATA;
        DIMENSIONS NTAX=88 NCHAR=72;
        FORMAT MISSING=X GAP=- DATATYPE=PROTEIN;
MATRIX
Tc_eve     IRRYRTAFTREQLARLEKEFFKENYVSRPRRCELAAQLNLPESTIKVWFQNRRMKDKRQRMAIAWPYAAVYT [72]
Bf_Mox     PRKERTAFTKQQIMELENEFRHHNYLTRLRRYEIAVKLDLTERQVKVWFQNRRMKWKRTKGGAAMRDKEALK [72]
Fen_Mox    PRKERTAFTKQQIKALEKEFNQHNYLTRLRRYEIAVALDLTERQVKVWFQNRRMKWKRVKGSQIAKDKVNQP [72]
Nv_Hox7    RKRGRQTYTRYQTLELEKEFHFNRYLTRRRRIEIAHSLCLTERQIKIWFQNRRMKWKKENKIVDNGPPMHGC [72]
Es_antp    RKRGRQTYTRYQTLELEKEFHFNRYLTRRRRIEIAHALCLTERQIKIWFQNRRMKWKKENKAEMPGTENGKP [72]
Fen_Hox6   RKRGRQTYTRYQTLELEKEFHFNRYLTRRRRIEIAHALCLTERQIKIWFQNRRMKWKKEQKAALGVGMPGPG [72]
Tc_antp    RKRGRQTYTRYQTLELEKEFHFNRYLTRRRRIEIAHALCLTERQIKIWFQNRRMKWKKENKTKGEGGSEGGG [72]
Lin_ant   RKRGRHTYSRHQTLELEKEFHFNRYLTRRRRIEIAHALCLTERQIKIWFQNRRMKWKKENKGIELSRDSPP- [71]
Cc_Hox7    RKRGRQTYTRYQTLELEKEFHFNRYLTRRRRIEIAHALCLTERQIKIWFQNRRMKWKKENRQIEVLRQHTDD [72]
Fen_Hox7   RKRGRQTYTRYQTLELEKEFHFNRYLTRRRRIEMAHALCLTERQIKIWFQNRRMKEKKEKQKIEEMKVDGVS [72]
Bf_Hox8    RRRGRQTYSRYQTLELEKEFHFNKYLTRRRRIEIAHALGLTERQIKIWFQNRRMKLKKEAAMLCPPKAETET [72]
Es_lox4    RRRGRQTYSRFQTLELEKEFQYNNYLTRKRRIEVAHALNLSERQVKIWFQNRRMKLKKEKQQIREMNGACRS [72]
Nv_lox4    EERGRQTYSRYQTLELEKEFRFNHYLTRKRRMEIAHVLCLTEHQIKIWFQNRRMKLRKERLQIKELNRDITG [72]
Cc_lox4    RRRGRQTYSRYQTLELEKEFQFNHYLTRKRRIEIAHALCLTERQIKIWFQNRRMKLKKERQQIKDLNGLDGK [72]
Tc_abdA    RRRGRQTYTRFQTLELEKEFHFNHYLTRRRRIEIAHALCLTERQIKIWFQNRRMKLKKELRAVKEINEQARR [72]
Tc_Ubx     RRRGRQTYTRYQTLELEKEFHTNHYLTRRRRIEMAHALCLTERQIKIWFQNRRMKLKKEIQAIKELNEQEKQ [72]
Cc_lox2    RRRGRQTYTRYQTLELEKEFKFNRYLTRRRRIELSHMLCLTERQIKIWFQNRRMKEKKEIQAIKELNEKEKT [72]
Nv_lox2    RRRGRQTYTRYQTLELEKEFKFNRYLTRKRRIELSHMLCLTERQIKIWFQNRRMKEKKEIQAIKDLNAEREP [72]
Ls_Hox7    RKRGRQTYTRYQTLELEKEFHFNKYLTRRRRIEIAHALCLTERQIKIWFQNRRMKWKKENKQPVGIVCKEE [72]
Bf_Hox7    RKRGRQTYTRYQTLELEKEFHFNKYLTRRRRIEIAHALCLTERQIKIWFQNRRMKWKKENKLESLKQQPAES [72]
Fen_Hox5   QKRTRQTYTRHQTLELEKEFHFNRYLTRRRRIEIAHALGLTERQIKIWFQNRRMKWKKENNLKSINDAKPEF [72]
Es_lox5    QKRTRQTYTRFQTLELEKEFHFNRYLTRRRRIEIAHSLGLSERQIKIWFQNRRMKWKKENNVSKLTGPDKSK [72]
Nv_lox5    QKRTRQTYTRYQTLELEKEFHYNRYLTRRRRIEIAHALGLTERQIKIWFQNRRMKWKKENNLSKLTGPNGEP [72]
Cc_lox5    QKRTRQTYTRYQTLELEKEFHYNRYLTRRRRIEIAHALQLTERQIKIWFQNRRMKYKKENNISKLTGPNGEL [72]
Bf_Hox6    KKRGRQTYTRYQTLELEKEFHFNKYLTRKRRIEIAHLLGLTERQIKIWFQNRRMKWKKENKIPSLNATTINQ [72]
Dj_plox6   HKRSRQTYTRYQTLELEKEFHFNKYLTRRRRIEIAHALSLTERQIKIWFQNRRMKWKKDHNIPKLNGPGTLE [72]
Tc_Scr     TKRQRTSYTRYQTLELEKEFHFNRYLTRRRRIEIAHALCLTERQIKIWFQNRRMKWKKEHKMASMNIVPYHM [72]
Es_scr     SKRSRTSYTRHQTLELEKEFHYNKYLTRRRRIEIAHALNLTERQIKIWFQNRRMKWKKEHKLSHIAKNMNVC [72]
Nv_Scr     SKRTRTSYTRHQTLELEKEFHFNRYLTRRRRIEIAHALNLTERQIKIWFQNRRMKWKKEHKLGHLAKSQGTK [72]
Cc_Scr     NKRTRTSYTRHQTLELEKEFHFNRYLTRRRRIEIAHSLNLTERQIKIWFQNRRMKWKKEHKLAHLAKSQAQK [72]
Fen_Hox4   PKRARTAYTRHQVLELEKEFHFNRYLTRRRRIEIAHALCLTERQIKIWFQNRRMKWKKDHGLPNTKTVKSRA [72]
Bf_Hox4    TKRSRTAYTRQQVLELEKEFHFNRYLTRRRRIEIAHSLGLTERQIKIWFQNRRMKWKKDNRLPNTKTRSSSA [72]
Tc_Dfd     PKRQRTAYTRHQILELEKEFHYNRYLTRRRRIEIAHTLVLSERQIKIWFQNRRMKWKKDNKLPNTKNVRRKT [72]
Nv_Dfd     SKRTRTAYTRHQVLELEKEFHFNRYLTRRRRIEIAHALCLTERQIKIWFQNRRMKWKKENKLPNTKNRLSNN [72]
Cc_Dfd     SKRTRTAYTRHQILELEKEFHFNRYLTRRRRIEIAHTLCLSERQIKIWFQNRRMKWKKEHKLPNTKTRLTDS [72]
Bf_Hox5    NKRTRTAYTRYQTLELEKEFHFNRYLTRRRRIEIAHALCLTERQIKIWFQNRRMKWKKENKLKSLSQCQQTQ [72]
Sr_Cent    CKRTRTAYTRFQTLELEKEFHFNRYLTRRRRIEIANLLALTERQIKIWFQNRRMKWKKDNNLKSMSQIDSIT [72]
Tc_ftz     NKRTRQTYTRYQTLELEKEFHFNKYLTRRRRIEIAESLRLTERQIKIWFQNRRMKAKKDTKFTEQSVTSTFD [72]
Fen_MP     HRKRRQTYTRHQTAELEREYVTNRYLTRRRRIEISQSLHLSERQIKIWFQNRRMKEKREEDHVTSPRKNKKK [72]
Sc_MP      HRKRRQTYSRQQTAELEREYVSNRYLTRRRRIEISQHLDLSERQIKIWFQNRRMKERREKDHPATTTTSPRK [72]
Bf_Hox9    SRKKRCPYTRFQTLELEKEFLYNMYLTRERRYEISQHVNLTERQVKIWFQNRRMKMKKMS------------ [60]
Sr_Post    SRKKRRPYPKNQTLELEKEFLFNTYITRERRLEIARSLNLTDRQVKIWFQNRRMKNKKQHSGGPGMGPPIPG [72]
Bf_Hox12   SRKKRCPYSKVQLLELEKEFLYNMYITREQRGEIARKVNLTDRQVKIWFQNRRMKMKRMKQRHEEE------ [66]
Bf_Hox10   GRKKRCPYTKYQILELEKEFLFNMYVSRERRQEISRHVNLSDRQVKIWFQNRRMKMKRMN------------ [60]
Bf_Hox11   TRKKRCPYTKYQTLELEKEFLFNMFVTRERRQEIARQLNLTDRQVKIWFQNRRMKMKRMKQRAM-------- [64]
Sr_cdx     KDKYRVVYTDRQRAELENEFRSAQYITIRRKSELAMQVGLSERQVKIWFQNRRAKERKVSRKVPGGGNHSSS [72]
Cc_Cdx     KDKYRIVYSEYQKVELEKEYLYSKYITIQRKAELSRSIGLSERQVKIWFQNRRAKERKQKRKMEEALSTSDS [72]
Nv_cdx     KDKYRVVYTDHQRLELEKEFHYSRYITIRRKAELAQNLNLSERQVKIWFQNRRAKERKQNKKREDGMQGAGS [72]
Tc_cdx     KDKYRVVYTDHQRVELEKEFYYSRYITIRRKAELANSLGLSERQVKIWFQNRRAKERKQVKKREEVTQKDSP [72]
Fen_cdx    KDKYRVVYSDHQRIELEKEYLQSPYITIKRKAELAQTLDLSERQVKIWFQNRRAKERKQFKKDSSASDSGKD [72]
Bf_cdx     KDKYRVVYSDHQRLELEKEFYSNKYITIKRKVQLANELGLSERQVKIWFQNRRAKQRKMA------------ [60]
Cc_Post1   PKKKRKPYSKPQVSALENEYSTSTYITKARRKEVARELDLTERQIKIWYQNRRIKEKKIATKRAKVQSDLFM [72]
Tc_AbdB    VRKKRKPYSKFQTLELEKEFLFNAYVSKQKRWELARNLNLTERQVKIWFQNRRMKNKKNSQRQAAQQQNNNN [72]
Bf_Hox13   GRKKRCPYTKYQLSVLEQEYIQNRYVSRETRLELSQRLNLTDRQVKIWFQNRRMKQKRLE------------ [60]
Bf_Hox14   VRPKRRPYSKYQLNELENEYVQNQYISRDKRLQLSQKLNLTERQVKIWFQNRRIKQKKLD------------ [60]
Es_post1   LRKRRRPYSKYQIAELEREYALSTYISKSRRWELSQLLNLSERQIKIWFQNRRIKAKKLQKRDETLKTQTPN [72]
Nv_Post1   MRKKRKPYSKYQIAELEKEYVNNTYITKPKRWELSQRLNLSERQVKIWFQNRRMKEKKVTDKKC-------- [64]
Es_post2   GRKKRKPYTRYQTMVLENEFLNSSYITRQKRWEISCKLQLTERQVKIWFQNRRAKERKKLNERAKARLREDRE [72]
Nv_post2   QRKKRKPYTRYQTMVLENEFMGNSYITRQKRWEISCKLHLSERQVKVWFQNRRMKRKKLNERAKTLIKS--- [69]
Cc_Post2   QRKKRKPYTRYQTMVLENEFINNSYITRQKRWEISCKLHLSERQVKVWFQNRRMKRKKLNERAKSLIKDHEP [72]
Fen_PostB CRTKRKPYEKWVTYLLEEEYLSNTYITKQKRYELSYRTSLTERQVKIWFQNRRMKSKKLRERSTSGATGNED [72]
Fen_PostA IRKKRKPYTKHQTFILEQEYLMSTYITRQRRLELARNLSLTERQVKIWFQNRRMKTKKLRERNKVSVGPDST [72]
Cc_Xlox    NKRTRTAYTRAQLLELEKEFHFNRYITRPRRVELAAHLNLTEQHIKIWFQNRRMKWKKDVDKKRPQQSEQDG [72]
Bf_Xlox    NKRTRTAYTRGQLLELEKEFHFNKYISRPRRIELAAMLNLTERHIKIWFQNRRMKWKKEQ------------ [60]
Nw_xlox    --------------------HFNKYISRPRRIELAAMLNLTERHIKIWFQNRRMKWKKDEAKRRPRPLKSGS [52]
Es_xlox    NKRTRTAYTRGQLLELEKEFHFNKYISRPRRIELAAMLNLTERHIKIWFQNRRMKWKKDEAKRRPRPLSTST [72]
```

```
Sr_labi    TRGGRTNFTNKQLTELEKEFHFNRYLTRARRIEIATSLTLNETQVKIWFQTRRMKQKKLLKEGKLS------ [66]
Fen_Hox1   NNTGRTNFTTKQLTELEKEFHFNKYLTRARRIEIAGALQLNETQVKIWFQNRRMKQKKRMKEGLIPPDPALT [72]
Tc_lab     LNTGRTNFTNKQLTELEKEFHFNKYLTRARRIEIASALQLNETQVKIWFQNRRMKQKKRMKEGLIPPEPISA [72]
Es_lab     NSTGRTNFTNKQLTELEKEFHFNKYLTRARRIEIAAALGLNETQVKIWFQNRRMKQKKRLKEAQGTPGLGNT [72]
Bf_Hox1    PNNGRTNFTTKQLTELEKEFHYNKYLTRARRVEIAAALNLNETQVKIWFQNRRMKQKKREKENG-------- [64]
Nv_lab     PNMGRTNFTNKQLTELEKEFHFNKYLTRARRIEIAAALGLNETQVKIWFQNRRMKQKKRMKETNVSPTF--- [69]
Cc_lb      PNMGRTNFTNKQLTELEKEFHFNKYLTRARRIEIAASLGLNETQVKIWFQNRRMKQKKRLKENTSTTPVSDS [72]
Tc_hox3    GKRARTAYTSAQLVELEREFHHGKYLSRPRRIQIAENLNLSERQIKIWFQNRRMKHKKEQMNKVSTPRSSPA [72]
Bf_Hox3    GKRARTAYTSAQLVELEKEFHFNRYLCRPRRVEMAAMLNLTERQIKIWFQNRRMKYKKEQKVKGGGSGGGSG [72]
Es_hox3    AKRARTAYTSAQLVELEKEFHFNQYLCRPRRIEMAALLNLSERQIKIWFQNRRMRFKKEKKLKVNMDKSGCG [72]
Nv_hox3    SKRARTAYNSAQLVELEKEFHFNRYLCRPRRIEMAALLSLSERQIKIWFQNRRMKYKKDQRMKPNSEKE--- [69]
Cc_Hox3    SKRARTAYTSAQLVELEKEFHFNRYLCRPRRIEMAALLNLTERQIKIWFQNRRMKYKKDQKQKNLMEKQYAG [72]
Bf_gsx     SRRMRTAFSSTQLLELEREFASNMYLSRLRRIEIATFLNLSEKQVKIWFQNRRVKHKKEA------------ [60]
Cc_Gsx     VKRMRTAFSSTQLLELEREFASNMYLSRLRRIEIATYLSLSEKQVKIWFQNRRVKFKKEGAAHGSRDHPHCQ [72]
Tc_ind     SKRIRTAFTSTQLLELEREFASNMYLSRLRRIEIATCLRLSEKQVKIWFQNRRVKYKKEDLPAAAGKASNGN [72]
Nv_gsx     GKRIRTAFTSTQLLELEREFSSNMYLSRLRRIEIATYLNLSEKQVKIWFQNRRVKYKKEGVTDSRDKCRCLR [72]
Es_gsx     SKRIRTAFTSTQLLELEREFASNMYLSRLRRIEIATYLNLSEKQVKIWFQNRRVKYKKEGTGESGEKCRCLR [72]
Bf_Hox2    SRRLRTVFTNTQLLELEKEFHYNKYVCKPRRKEIASYLDLNERQVKIWFQNRRMRQKRRDTKGR-------- [64]
Cc_pb      PRRLRTAYTNTQLLELEKEFHFNKYLCRPRRIEIAASLDLTERQVKVWFQNRRMKFKRQTGKGSGNSPDAEC [72]
Nv_pb      PRRLRTAYTNTQLLELEKEFHFNKYLCRPRRIEIAASLDLTERQVKVWFQNRRMKFKRQTQTKGLPDGNSDD [72]
Tc_pb      PRRLRTAYTNTQLLELEKEFHFNKYLCRPRRIEIAASLDLTERQVKVWFQNRRMKHKRQTLGKQGDDGDDKD [72]
Bf_EVX     VRRYRTAFTREQLARLEKEFYRENYVSRPRRCELAAQLNLPETTIKVWFQNRRMKDKRQRLALTWPHPADPN [72]
;
END;
```
