# Supplementary figures and images for: Genomic Organization and Expression Demonstrate Spatial and Temporal Hox Gene Colinearity in the Lophotrochozoan Capitella sp. I
Source: PLoS One. 2008 Dec 23;3(12):e4004. doi: 10.1371/journal.pone.0004004 (PMC2603591; doi:10.1371/journal.pone.0004004)

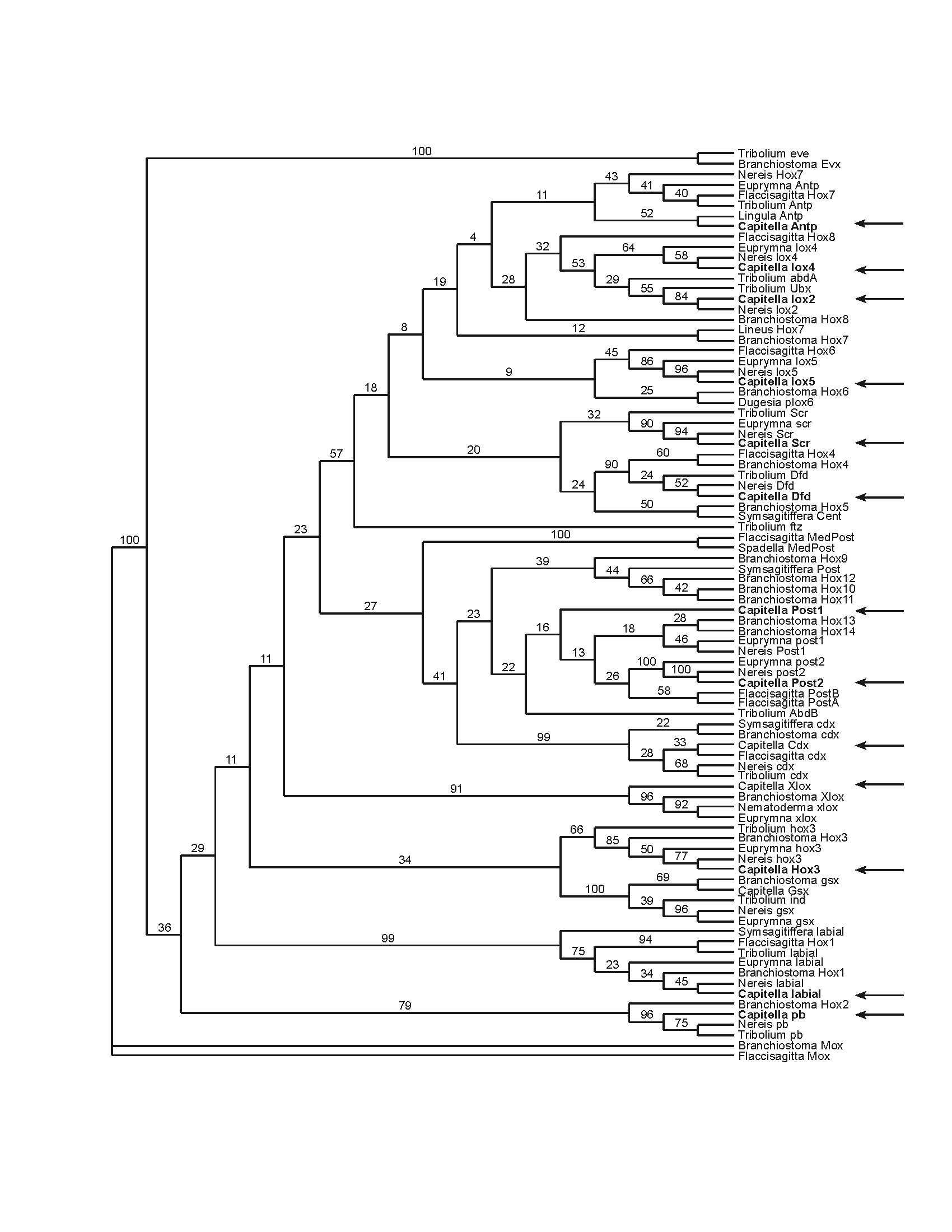

Supplement: Figure S2 — Neighbor-joining bootstrap consensus tree. A neighbor-joining (NJ) bootstrap consensus tree (using mean amino acid distances) was constructed using PAUP* v4.0b10 [29] with 1,000 iterations, using a 72-AA alignment of representative bilaterian Hox and Parahox genes (see Figure S1), including the 60-AA homeodomain as well as the 12 AAs immeditately flanking the 3′ end of the homeodomain. Numbers above branches indicate NJ bootstrap support, shown as a percentage. New Capitella sp. I sequences are shown in bold; all Capitella sequences are delimited by an arrow. (0.39 MB TIF) [file pone.0004004.s002.tif]

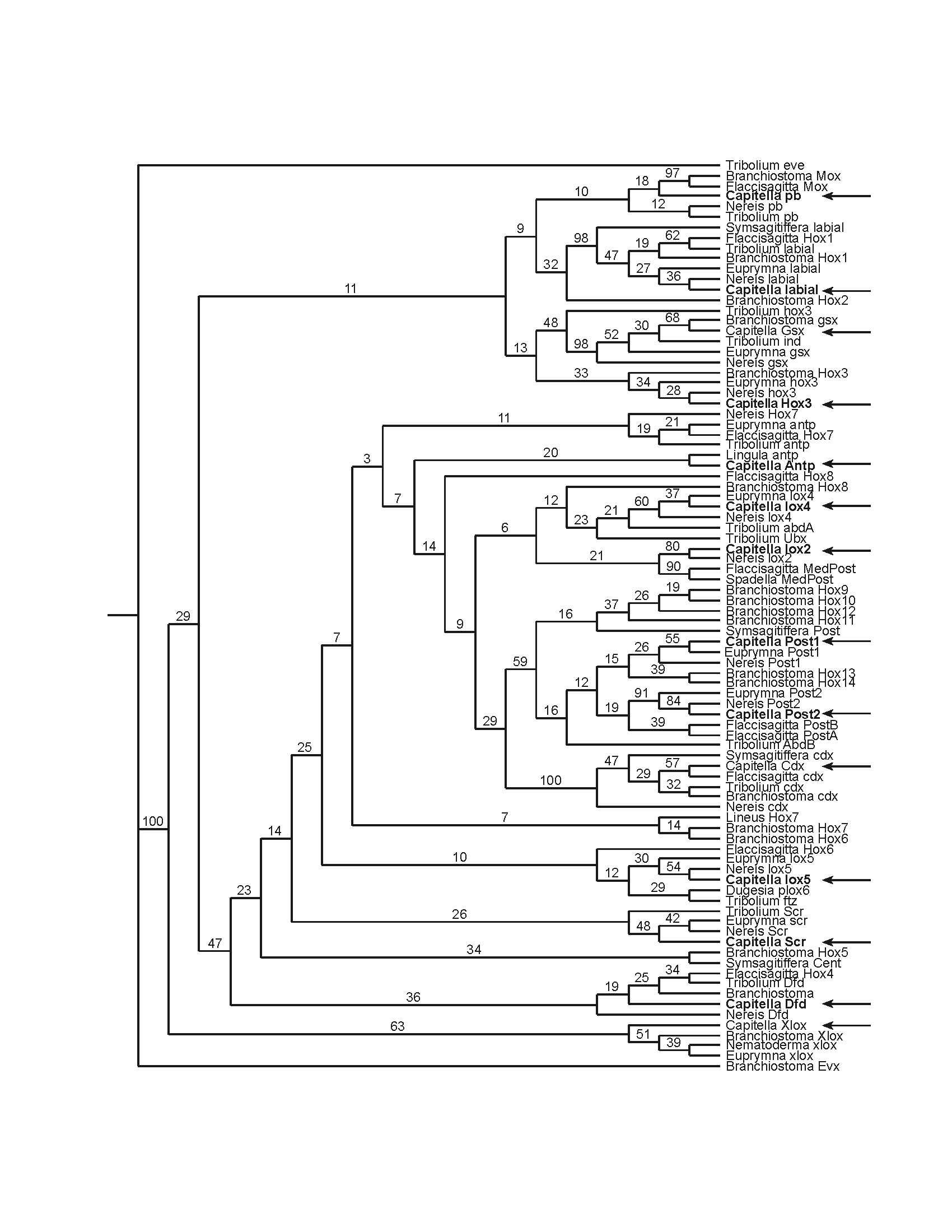

Supplement: Figure S3 — Maximum likelihood bootstrap consensus tree. A maximum likelihood (ML) bootstrap consensus tree was constructed using RAXML v2.2.1 [31] using the rtrev+G model of protein evolution, selected via ProtTest [30]. An initial search of 500 iterations was conducted to determine consistency of recovering the most likely tree (unpublished data). An additional 1,000 bootstrap iterations were conducted in RAXML v2.2.1. Numbers above branches indicate ML bootstrap support shown as a percentage. New Capitella sp. I sequences are shown in bold; all Capitella sequences are delimited by an arrow. (0.40 MB TIF) [file pone.0004004.s003.tif]
